# Mapping the global distribution of C_4_ vegetation using observations and optimality theory

**DOI:** 10.1038/s41467-024-45606-3

**Published:** 2024-02-09

**Authors:** Xiangzhong Luo, Haoran Zhou, Tin W. Satriawan, Jiaqi Tian, Ruiying Zhao, Trevor F. Keenan, Daniel M. Griffith, Stephen Sitch, Nicholas G. Smith, Christopher J. Still

**Affiliations:** 1 Department of Geography, National University of Singapore, Singapore, Singapore; 2 Center for Nature-based Climate Solutions, National University of Singapore, Singapore, Singapore; 3 School of Earth System Science, Institute of Surface-Earth System Science, Tianjin University, Tianjin, China; 4 Department of Ecosystem Sciences, Policy and Management, UC Berkeley, Berkeley, CA USA; 5 Earth and Environmental Sciences Area, Lawrence Berkeley National Lab, Berkeley, CA USA; 6 Department of Forest Ecosystems and Society, Oregon State University, Corvallis, OR USA; 7 Faculty of Environment, Science and Economy, University of Exeter, Exeter, UK; 8 Department of Biological Sciences, Texas Tech University, Lubbock, TX USA

**Keywords:** Climate and Earth system modelling, Ecophysiology, Ecosystem ecology

## Abstract

Plants with the C_4_ photosynthesis pathway typically respond to climate change differently from more common C_3_-type plants, due to their distinct anatomical and biochemical characteristics. These different responses are expected to drive changes in global C_4_ and C_3_ vegetation distributions. However, current C_4_ vegetation distribution models may not predict this response as they do not capture multiple interacting factors and often lack observational constraints. Here, we used global observations of plant photosynthetic pathways, satellite remote sensing, and photosynthetic optimality theory to produce an observation-constrained global map of C_4_ vegetation. We find that global C_4_ vegetation coverage decreased from 17.7% to 17.1% of the land surface during 2001 to 2019. This was the net result of a reduction in C_4_ natural grass cover due to elevated CO_2_ favoring C_3_-type photosynthesis, and an increase in C_4_ crop cover, mainly from corn (maize) expansion. Using an emergent constraint approach, we estimated that C_4_ vegetation contributed 19.5% of global photosynthetic carbon assimilation, a value within the range of previous estimates (18–23%) but higher than the ensemble mean of dynamic global vegetation models (14 ± 13%; mean ± one standard deviation). Our study sheds insight on the critical and underappreciated role of C_4_ plants in the contemporary global carbon cycle.

## Abstract (web summary)

Due to fundamental anatomical and biochemical differences, C_3_ and C_4_ plant species tend to differ in their biogeography and response to climate change. Here, the authors use global observations and optimality theory to map patterns and temporal trends in C_4_ species distribution and the contribution of C_4_ plants to global photosynthesis.

## Introduction

C_4_ is one of the three photosynthetic pathways for terrestrial plants^1^ and is reported to account for 18–23%^2–4^ of global photosynthesis. C_4_ plants also drive wildfire dynamics in tropical and subtropical ecosystems^5^. C_4_ plants first evolved in the low atmospheric CO_2_ environment of the Oligocene Epoch, roughly 24–35 million years ago^6^. They developed distinct biochemical and anatomical characteristics to enrich CO_2_ concentration at the site of Rubisco carboxylation in leaves, thereby reducing photorespiration and enhancing carbon-fixation rates^7^. These characteristics produce different climate sensitivities in C_4_ plants compared to more prevalent C_3_ plants^4,8^, and thus are expected to cause a shift in C_4_ plant distributions and their contribution to global photosynthesis under contemporary and future climate change^8–10^.

Many previous studies have examined C_4_ plant responses to multiple environmental factors. A consensus is that since most C_4_ species originated in lower atmospheric CO_2_ concentrations^11,12^, they are expected to benefit less from rising CO_2_ concentrations compared to C_3_ plants. Meanwhile, higher temperatures are expected^4^ and reported^13–15^ to favor C_4_ over C_3_ photosynthesis, because the affinity of O_2_ to Rubisco relative to CO_2_ becomes stronger with increasing temperature and also due to differing solubilities of CO_2_ and O_2_ with increasing temperature. This should produce an advantage for the carbon concentrating mechanism of C_4_ species, especially under high temperatures^16^. Hence C_4_ species are characteristic of tropical and subtropical ecosystems. Correspondingly, since C_4_ photosynthesis is less limited by CO_2_ than C_3_ photosynthesis, it achieves a higher photosynthetic quantum yield and photosynthetic rates under high light, especially under high temperatures^17^. C_4_ species also should have a carbon assimilation advantage in arid environments^18,19^ due to their higher water use efficiency (i.e., less water loss through stomata for equivalent carbon gain) than C_3_ species, though under humid conditions this advantage could be limited^9^. Contemporary climate change, such as elevated CO_2_, rising temperatures, and changing rainfall patterns, can therefore lead to temporal and spatial shifts in the relative advantages of C_4_ to C_3_ photosynthesis. For instance, the differential response to a changing environment has been linked to observed woody plant encroachment in tropical Africa, where an increase in precipitation and elevated atmospheric CO_2_ levels are hypothesized to have caused a net decrease in C_4_ grassland distribution^20,21^. However, we currently lack a consensus on how the relative advantages of C_4_ to C_3_ photosynthesis change at the global scale, as regional studies have reported contrasting results and different driving factors—such as increased C_4_ grass distribution due to increased temperature^22^, decreased distribution due to elevated CO_2_^14^ or no overall trend^23^. Understanding of how climate change has impacted C_4_ vegetation constitutes a major challenge due to its role in global photosynthesis and the terrestrial carbon cycle.

C_4_ vegetation overwhelmingly consists of natural grasses and crops using the C_4_ pathway. One prominent approach to estimate the distribution of C_4_ natural grasses is based on the crossover-temperature model, which predicts that a particular month is determined to favor C_4_ grasses over co-occurring C_3_ grasses when the mean daytime air temperature is >22 °C and precipitation in that same month is ≥25 mm^2,24,25^. This approach is based on each pathway’s relative carbon assimilation as a function of temperature, and thus the crossover temperature is dependent on atmospheric CO_2_ concentration with higher crossovers at higher CO_2_ levels. A few efforts to model C_4_ vegetation distribution have further incorporated the seasonality of precipitation^26–29^, or mean annual temperature and precipitation^8,22,26^, but so far they are only validated and applied at the regional scale. Some dynamic global vegetation models (DGVMs) allow adjustment of C_3_ and C_4_ grass distribution based on the difference between simulated C_3_ and C_4_ photosynthesis or the difference between their net primary productivity^30^, or based on the simulations from bioclimate distribution models in each time step, with the baseline C_4_ map acquired from remote sensing land cover classifications^31,32^. Some cohort-based DGVMs further consider competition for resources^33^ and disturbances^34^ when simulating C_4_ distributions. In general, current estimates of the distribution of C_4_ vegetation adopt a wide range of assumptions and generate rather different results^10^.

Uncertainty in global C_4_ grass distribution is further exacerbated by the lack of ground observations for validation and then for model extrapolation, since previous models often relied on either local datasets^26,27^ or literature reviews of C_4_ grass presence and absence^2^ for validation. This issue has become less prominent recently as some studies have used continental scale (i.e., North America) C_4_ plots^25^ and ^13^C isotopic records^23,35^ to validate C_4_ grass distribution models. Meanwhile, the distribution of C_4_ crops has been collated and estimated in some open datasets^36–39^. These datasets are based on Food and Agriculture Organization (FAO) census and national reporting of the harvested area for major C_4_ crops (i.e., maize, sorghum, millet, sugarcane), which comprised 24% of the global harvested area^37^, and are supplemented by total cropland area change from FAO and remote sensing^40^. In particular, the Land-Use Harmonization dataset version 2 (LUHv2) is the principal gridded land use dataset for the assessment of global carbon budgets^36^ and future climate change in CMIP6^41^, in which C_4_ crop area over time is explicitly reported. Changes in global C_4_ crop distribution and the related contribution to global photosynthesis have yet to be evaluated, except for a few studies that have examined the C_4_ crop distribution for specific years^2,42,43^,

Here we quantify the global C_4_ vegetation distribution (including natural grasses and crops) and its contribution to global photosynthesis, as well as examine changes in C_4_ vegetation distribution over the past two decades. To do so, we use photosynthetic optimality theory to estimate the relative advantage of C_4_ to C_3_ photosynthesis over the global land surface, and then use the estimated difference in combination with observations to infer global C_4_ grass distribution. The optimality model includes a wide array of selective drivers for C_4_ grass distribution - CO_2_, temperature, light, aridity, nitrogen, and their interactions^44^ (see Methods), which are advances over previous crossover-temperature approaches which include CO_2_, temperature, and a precipitation threshold. The optimality model estimates the optimal leaf photosynthetic rate for C_3_ and C_4_ plants, along with optimal stomatal conductance and root/shoot carbon allocation based on growing season climate, with a target to maximize carbon gain with minimized water loss^44^. We further take advantage of multiple open-access databases of C_4_ species richness and coverage (i.e., the global TRY database^45^, a dataset for the contiguous United States (the DG dataset)^23^ and a subset of the Nutrient Network (NutNet)^46^), global grassland fraction maps from remote sensing (i.e., as the majority of C_4_ plant cover is non-woody^47^), in combination with the optimality model simulations to acquire data-constrained estimates of C_4_ grass distribution for the past 20 years. Meanwhile, we obtain and examine C_4_ crop distribution using multiple open datasets^36,39^. We further use an emergent constraint technique—a method to infer an unobservable variable from an observable variable based on the large spread of estimates of both variables from DGVMs (see Methods) –to estimate the contribution of C_4_ plants to global photosynthesis. By quantifying how C_4_ vegetation distribution and photosynthesis have changed over recent decades, our study improves understanding of historical changes in terrestrial photosynthesis and the global carbon cycle.

## Results

### C_4_ photosynthetic advantage and C_4_ grass coverage

**Fig. 1:**
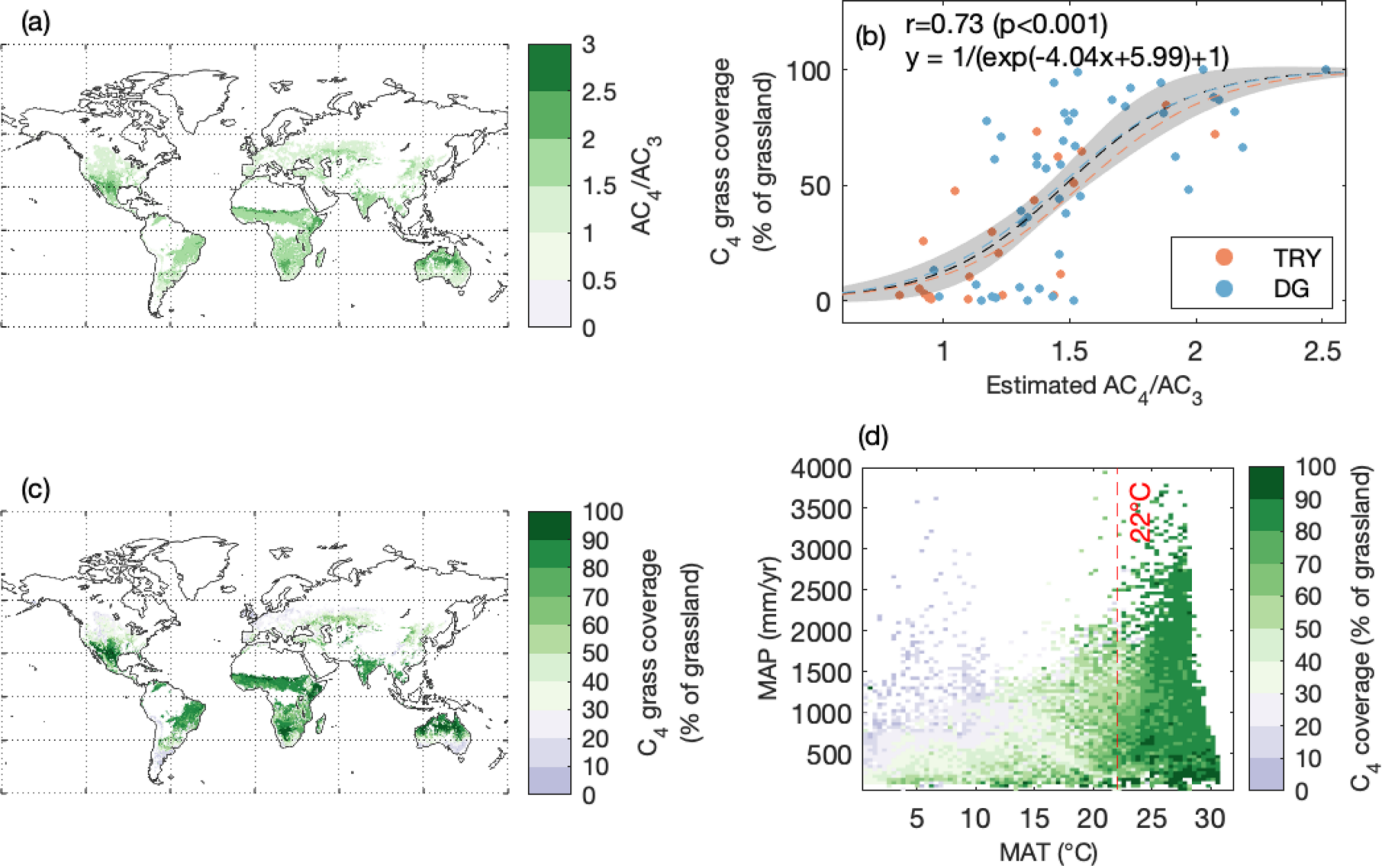
C_4_ natural grass coverage estimated by the optimality model. **a** the ratio of C_4_ to C_3_ photosynthesis estimated by the optimality model (AC_4_/AC_3_) over global non-woody regions; (**b**) the relationship between observed C_4_ coverage (% of grassland) and estimated C_4_/C_3_ photosynthetic ratio by the optimality model. C_4_ coverage observation obtained from difference sources (i.e., TRY, DG datasets; please see methods); gray shaded area indicates the uncertainty range for the relationship between AC_4_/AC_3_ and C_4_ coverage (i.e., 95% confidence interval). The black line represents the regression using both the TRY and DG datasets, while the red and blue dash lines represent the regression using either the TRY or the DG dataset. **c** C_4_ grass coverage (% of grassland) over the globe, which can be regarded as the potential C_4_ area abundance when grassland covers 100% of the land surface; (**d**) C_4_ coverage in a climate space of mean annual temperature (MAT: °C) and mean annual precipitation (MAP: mm/yr).

We found a strong positive relationship between the observed C_4_ grass coverage (i.e., the % of grassland area covered by C_4_ grass species) and the relative advantage of C_4_ photosynthesis (AC_4_) over C_3_ photosynthesis (AC_3_) estimated by the optimality model (denoted as the AC_4_/AC_3_ - C_4_ coverage relationship hereafter; see Methods; Fig. 1b). With the increase in modeled AC_4_/AC_3_, the observed C_4_ coverage increased and then gradually plateaued. When AC_4_/AC_3_ = 1, C_4_ accounts for only 5.2% of grassland cover; when AC_4_/AC_3_ = 2.5, C_4_ coverage approaches 100% (Fig. 1b). Across global non-woody regions, AC_4_/AC_3_ ranged from 0.5 to 2.5, with a mean of 1.9 (Fig. 1a). Importantly, we obtained C_4_ coverage observations from multiple sources (i.e., TRY and DG; see Methods), which have different geographic representations and spatial resolutions. However, the AC_4_/AC_3_ - C_4_ coverage relationships are similar when using different observations (Fig. 1b), affirming the robustness of the relationship for C_4_ coverage estimation. Using the relationship between AC_4_/AC_3_ and C_4_ coverage (Fig. 1b), and the global AC_4_/AC_3_ estimated from the optimality model (Fig. 1a), we estimated the global C_4_ grass coverage (% of grassland covered by C_4_; Fig. 1c). We found C_4_ grass coverage followed a clear climatic gradient, and tended to be greater under warmer conditions (Fig. 1d).

### The global distribution of C_4_ vegetation

**Fig. 2:**
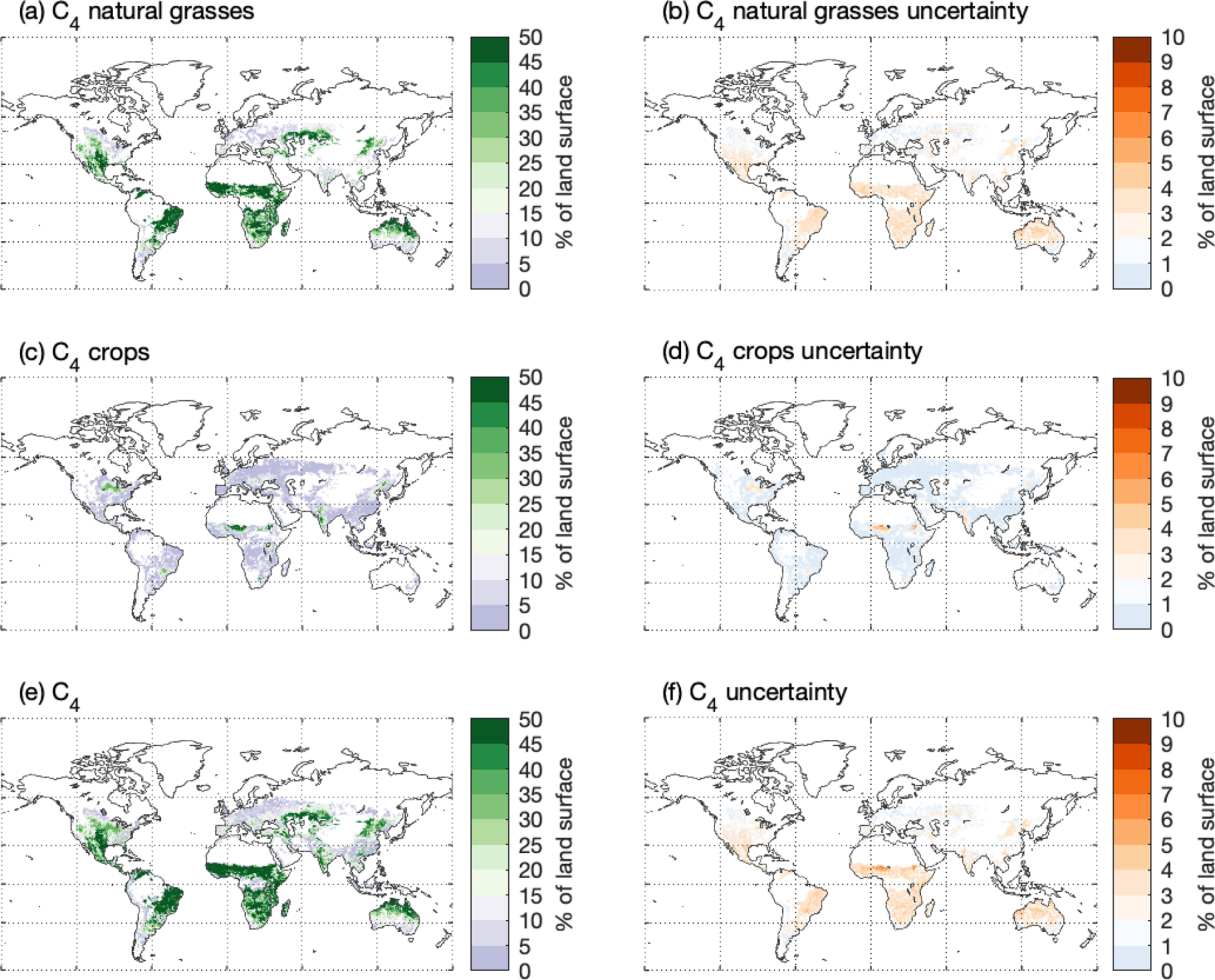
The modeled global distribution of C_4_ vegetation and associated uncertainties. The area occupied by (**a**) C_4_ natural grasses, (**c**) C_4_ croplands and (**e**) all C_4_ vegetation (unit: % of the land surface). The uncertainties of the area abundance of (**b**) C_4_ natural grasses, (**d**) C_4_ croplands and (**f**) all C_4_ vegetation (unit: % of the land surface).

After predicting the C_4_ grass coverage (% of grassland covered by C_4_ grasses; Fig. 1c), we overlaid a global grassland fraction map from remote sensing (see Methods and Fig. S1) to estimate the actual C_4_  natural grass area abundance (% of the land surface covered by C_4_ grasses; Fig. 2a). From 2001 to 2019, C_4_ natural grass accounted for 14.8 ± 1.3% (mean ± one standard deviation) of the non-frozen land surface area (Fig. 2a), while C_4_ crops accounted for 2.8 ± 0.3% (Fig. 2c). The total estimated C_4_ area abundance was 17.5 ± 1.4% (Fig. 2e). There were several C_4_ natural grass hotspots (i.e., >30% C_4_ area abundance) across continents (Fig. 2a): the Great Plains in North America, the savannas in Southern Brazil, the savannas in Africa, the grasslands in Central Asia, and Northern Australia. Meanwhile, we found the main C_4_ crop zones were in central North America, the Sahel region, and the west coast of India (Fig. 2c). The disagreement between remote sensing-based grassland fraction maps (Fig. S4), along with the uncertainty in the AC_4_/AC_3_ - C_4_ coverage relationship (Fig.1b), incurred uncertainties in the C_4_ natural grass distribution (Fig. 2b)—the uncertainty typically ranges between 1 and 3% of the land surface area, though in regions like Australia the uncertainty could be as high as 6–7% (Fig. 2b).

### The changes in C_4_ vegetation distribution

**Fig. 3:**
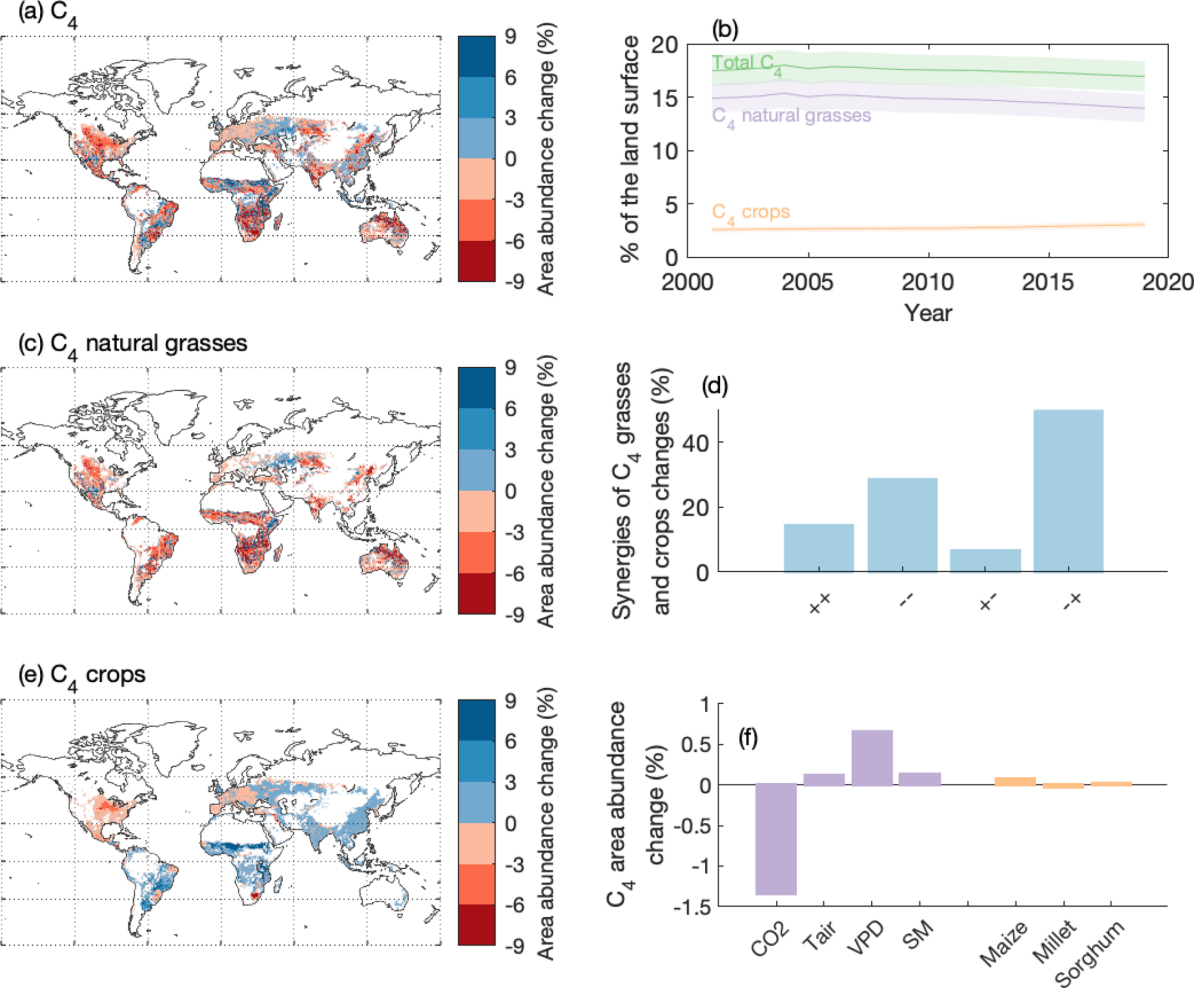
Changes in the global distribution of C_4_ vegetation between 2001–2019. Spatial distributions of changes in (**a**) total C_4_ vegetation, (**c**) C_4_ natural grasses and (**e**) C_4_ crops from 2001 to 2019; **b** The changes in the total area of C_4_ vegetation, C_4_ natural grasses and C_4_ crops, in percentages of global vegetated land surface; **d** the synergies of changes in C_4_ natural grasses and C_4_ crops, where ++ means both C_4_ natural grasses and C_4_ crops area abundance increased, − − means both decreased, + − means C_4_ natural grasses increased and C_4_ crops abundance decreased, − + means the opposite; **f** the drivers for the change in C_4_ natural grasses and C_4_ crops area abundances. Climate drivers include atmospheric CO_2_ concentration, air temperature (T_air_), vapor pressure deficit (VPD) and soil moisture (SM) during the growing seasons. In (**b**), the uncertainty for C_4_ crop is 10% of the C_4_ crop area reported, the uncertainty for C_4_ natural grass area is the combination of the uncertainty in remote sensing-based grassland fraction and the uncertainty of the AC_4_/AC_3_ - C_4_ coverage relationship (Fig. 1b), and uncertainty for C_4_ vegetation is the combination of uncertainties of C_4_ crop and C_4_ natural grass areas.

Based on our simulation of C_4_ natural grass distribution for the past two decades and the C_4_ cropland distribution from the LUHv2 dataset (see Methods), we found the overall area of C_4_ vegetation decreased from 17.7 ± 1.4% (mean ± one standard deviation) in 2001–2005 to 17.1 ± 1.4% in 2015–2019, as a net effect of a decrease in C_4_ natural grasses from 15.0 ± 1.3% to 14.2 ± 1.3%, and an increase in C_4_ crops from 2.6 ± 0.3% to 3.0 ± 0.3% (Fig. 3b). The change in C_4_ shows large spatial heterogeneity (Fig. 3a). In particular, C_4_ natural grass area decreased all over the globe, except for the central Europe and parts of the western U.S. (Fig. 3c). C_4_ crop area increased in most parts of the world, except for central North America where there was the largest decrease, and Europe where there were slight decreases (Fig. 3e).

The increase in C_4_ crop area was often at the expense of decreasing C_4_ natural grasses, as we found that in regions where there were both C_4_ crops and C_4_ natural grasses, more than 50% of the regions showed C_4_ natural grasses decreased but C_4_ crops increased. Meanwhile, only 14% showed that C_4_ crops and C_4_ natural grasses increased simultaneously, 28% showed they both decreased, and only 7% of the region showed that C_4_ natural grasses increased and C_4_ crops decreased (Fig. 3d). Our attribution analysis suggested that elevated CO_2_ was the dominant reason for the decrease in C_4_ natural grass distribution, while the impacts of temperature and water stress (i.e., soil moisture and vapor pressure deficit) were positive (i.e., increase C_4_ coverage) over the study period (Fig. 3f). Most of the increase in C_4_ crops, as we analyzed from another independent dataset on major C_4_ crop distributions^32^, came from the expansion of maize in South America and eastern Europe (Fig. 3f; Fig. S9; see Methods).

### The contribution of C_4_ vegetation to global photosynthesis

**Fig. 4:**
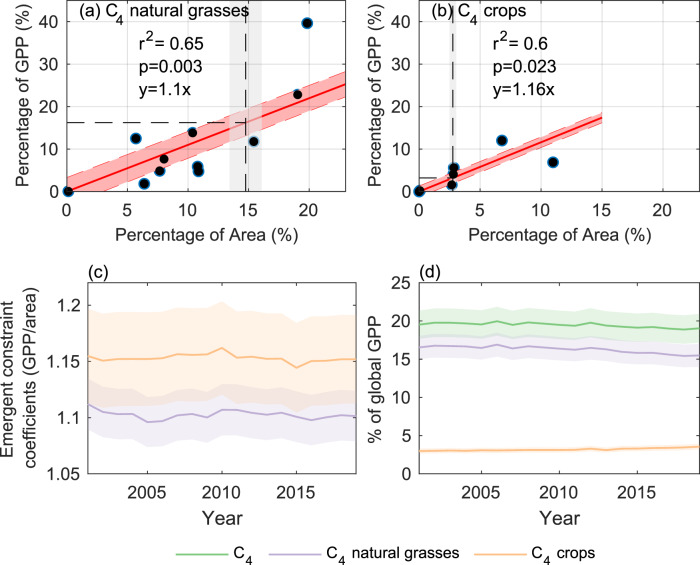
The contribution of C_4_ vegetation to global photosynthesis. The emergent constraints between the percentage of area occupied and the percentage of global photosynthesis contributed by (**a**) C_4_ natural grasses and (**b**) C_4_ crops, based on the estimates from an ensemble of the DGVMs; **c** changes in emergent constraint coefficients (i.e., the slopes of the linear regressions in (**a**, **b**) from 2001 to 2019 for C_4_ natural grasses and C_4_ crops). The uncertainties in (**a**, **b**, **c**) were quantified as one standard error (i.e., SE) by bootstrapping models when getting emergent constraint; **d** The contributions of C_4_, C_4_ natural grasses and C_4_ crops to global GPP from 2001 to 2019, whereas the uncertainty was quantified as one SE by bootstrapping the uncertainty range of C_4_ areas and the uncertainty range of the emergent constraint coefficients.

The changes in the C_4_ area can cause associated changes in total C_4_ photosynthesis, thus impacting global carbon cycle dynamics. Current ensemble of DGVMs predicted that C_4_ vegetation contributed from 2% to 40% of global photosynthesis, on 7% to 23% of the global vegetated land surface area (Figs. S6, S7). The large spread of model estimates indicates the various assumptions adopted in C_4_ vegetation distribution and potentially the different parameterizations for C_4_ photosynthesis in DGVMs. Despite these wide inter-model variations, it is possible to infer emergent constraints on C_4_ vegetation contributions to the carbon cycle. To quantify the contribution of C_4_ photosynthesis to global photosynthesis, we established an emergent constraint (*p* < 0.01) between the DGVM-simulated occupied area and percentage contribution of C_4_ natural grasses and crops to global photosynthesis, respectively. We found that with a 1% increase in area, C_4_ natural grass contribution to global photosynthesis increased by 1.10% (Fig. 4a), while the contribution of C_4_ crops increased by 1.16% (Fig. 4b). We also conducted a grid cell-level emergent constraint analysis and acquired similar ranges of slopes (Fig. S10). The lower coefficient of emergent constraint for C_4_ grass (i.e., 1.10) than the coefficient for C_4_ crop (i.e.,1.16) suggests that croplands tend to have a higher ecosystem photosynthetic rate compared to grasslands over the same area.

We further explored the changes in the coefficients of emergent constraints over the past two decades. We note that for C_4_ grasslands and C_4_ croplands, the coefficients all slightly decreased in the past two decades. The coefficient of C_4_ crops decreased from 1.16 to 1.15, while the coefficient of C_4_ grasses decreased from 1.11 to 1.10 from 2001 to 2019 (Fig. 4c). Interestingly, the coefficients are all greater than 1, highlighting that the per unit area photosynthetic rate of C_4_ is generally higher than that of the remaining C_3_ vegetation. With the likely increase of global photosynthesis in recent decades, the decreasing coefficients of C_4_ indicated that C_4_ photosynthesis increased at a slower pace than other (mostly C_3_) vegetation. By applying the estimated area of C_4_ to the annual emergent constraint coefficients (Fig. 4c), we found that the global C_4_ natural grass contribution to photosynthesis decreased from 16.5 ± 1.5% (mean ± one standard deviation) in 2001–2005 to 15.5 ± 1.5% in 2015–2019, the C_4_ crop contribution to photosynthesis increased from 3.0 ± 0.3% to 3.4 ± 0.4%, and in total the C_4_ contribution to global GPP decreased from 19.7 ± 1.9% to 19.0 ± 1.9% (Fig. 4d). The reported value is greater than the ensemble mean reported by the DGVMs (14 ± 13%), and is within the range of previously modeled values (18–23%^2–4^).

## Discussion

In this study, we estimated the global C_4_ vegetation distribution and quantified the changes in C_4_ vegetation distribution and photosynthesis over the past two decades, using an optimality photosynthesis model, photosynthetic pathway records from global/regional databases and remote sensing observations. On average, from 2001 to 2019, C_4_ plants occupied 17.5 ± 1.4% of the global vegetated surface and contributed 19.5 ± 1.9% of global photosynthesis, within the range of previous estimates (i.e., 18–23% for photosynthesis) but are greater than the estimates from the ensemble mean of DGVMs (13 ± 8% for area and 14 ± 13% for photosynthesis). C_4_ total area and C_4_ contribution to global photosynthesis both decreased over this period (i.e., 0.6% of the land surface and 0.7% of global GPP), which resulted from the increases in C_4_ crop area and its contribution to global photosynthesis, and the decreases in C_4_ natural grass area and its contribution to global photosynthesis.

Our study suggests that the decrease in C_4_ natural grass distribution was primarily driven by elevated CO_2_, in accordance with previous theoretical and experimental works that showed C_4_ advantage in carbon assimilation over C_3_ decreased with rising CO_2_^48–50^. There is also evidence showing many grassland and savanna areas have been invaded by C_3_ woody species, with increased atmospheric CO_2_ proposed as a major driver for the encroachment^51,52^. The decrease in the emergent constraint coefficients demonstrated that the impact of elevated CO_2_ on C_4_ and C_3_ were included in most DGVMs (Fig. 4c). Evidence for the historical expansion of C_4_ over geological time scales seems to support our conclusion, in particular for Africa where CO_2_ dominates the C_4_ grassland expansion or decline^50,53,54^, while in Central Asia^55^, Australia^56^, central China^57^ and central US grasslands^58^ reports show hydroclimatic change impacted C_4_ grass distribution. Our results also show that the changes in soil moisture and VPD over the past two decades largely induced positive impacts on C_4_ grass distribution, though globally their impacts were unable to offset the negative changes driven by elevated CO_2_ (Fig. 3f; Fig. S8).

To assess the distribution of C_4_ vegetation, we noted that there were three types of abundance used in previous literatures: for modeling practice, we often used the area abundance, that is, the percentage of the land surface occupied by C_4_ plants. However, the area abundance was challenging to observe over large scales, and it was rare to have direct observations of large-scale area abundance other than approximations from remote sensing^32^. Most observations of C_4_ presence and cover are at the species level and, therefore, observation-based studies often report the relative species richness of C_4_ plants^13,26^. A few studies reported biomass abundance, i.e., the percentage of local biomass contributed by C_4_ plants, and biomass abundance is often much higher than the species abundance^27,29^, echoing some studies suggesting that species abundance should be used with caution to infer biomass abundance and productivity^56^. In this study, we developed a conversion factor to translate C_4_ species richness into C_4_ area abundance, using plot-level concurrent measurements of both from NutNet. We found 1% increase in C_4_ species richness led to 1.51 ± 0.15% in C_4_ area abundance (Fig. S3). The conversion factor enabled us to translate global observations of C_4_ species richness into C_4_ coverage to develop the AC_4_/AC_3_ - C_4_ coverage relationship in our study (Fig. 1b, S1).

Compared to the previously estimated C_4_ vegetation distribution from a crossover-temperature model^2^ (Fig. S5), our study provided a similar area occupied by C_4_ vegetation (20.5 million km^2^ versus 21.1 million km^2^). We find a lower estimate for Africa, where our estimate of C_4_ area abundance showed a more nuanced gradient compared to the estimate from the crossover-temperature model (Fig. S5). We suspect this is partly due to their difference in relating C_4_ photosynthetic advantage to C_4_ grass coverage (% of grassland covered by C_4_ grasses) – while our study used the AC_4_/AC_3_ - C_4_ grass coverage relationship to gradually adjust C_4_ grass coverage, the crossover-temperature model assumes all grasslands in the pixel are C_4_ grassland as long as the monthly climate satisfies the crossover criteria (e.g., mean daytime air temperature is >22 °C and precipitation in that same month is ≥25 mm). Therefore, for regions where monthly climate meets the crossover criteria (e.g., sub-Sahel Africa), the crossover-temperature model tends to estimate 100% C_4_ grass, however, it was not the case for the optimality approach in those regions (Fig. 1c). Meanwhile, we estimated considerably higher C_4_ grass cover in Central Asia, which is consistent with the reported prevalence of C_4_ species in the region^26,59^ – the high abundance in these inland regions were potentially due to harsh environments characterized by high maximum temperature and aridity levels, which favor the growth of C_4_ plants over C_3_. We also note that our approach may underestimate C_4_ grass distribution in some mesic savanna ecosystems—such as the longleaf pine savannas in the Southeastern US—where a C_4_ understory exists beneath a C_3_ canopy^60,61^. The underestimation is likely because the optimality model predicted no photosynthetic advantage for C_4_ plants over C_3_ under low light conditions in understory (Fig. 1a), and the remote sensing products reported low grassland fraction in the region (Fig. S4).

In our study, we estimated the annual distribution of C_4_ grasses using the growing season mean climate; however, many locations have seasonal shifts between C_4_ and C_3_ grass dominance depending on seasonal climate variations^4^. For example, in the grasslands of southeast Australia, a recent study suggests C_4_ dominance is the highest in summer when there is high temperature and low precipitation, while other seasons have more C_3_ vegetation^28^. Therefore, for modeling the seasonal variation of carbon fluxes from seasonal changes in C_4_ grass distribution, the crossover-temperature approach based on monthly climate and weighted by a vegetation index like NDVI could be more useful^2^. We acknowledge that the distribution of our observations was not uniform across the globe, with North America being better represented compared to other regions (Fig. S2). As an additional test to validate our estimation of C_4_ vegetation distribution, we compared the C_4_ grass coverage estimated in our study (Fig. 1c) with the C_4_ coverage estimated from isotopic measurements and remote sensing in Australia^62^ (Fig. S11). This validation demonstrates a strong agreement (*r* = 0.69, *p* < 0.01) between the two independent estimates, affirming the robustness of our estimates in under-sampled regions.

We also need to highlight that the optimality approach was based on the assumption that C_4_ grass distribution is determined by the photosynthetic advantage of C_4_ compared to C_3_, and the photosynthetic advantage is largely dependent on local climate^44^. While this assumption is also adopted by the crossover-temperature model, it neglects the role of grass phylogeny in determining C_4_ grass distributions. Some studies have suggested that grass clades (i.e., Pooideae for C_3_, and PACCMAD for C_3_ and C_4_) are perhaps more critical than photosynthetic pathway for determining C_4_ and C_3_ grass distributions, at least along temperature gradients^19,63,64^. This lack of consideration on phylogeny in C_4_ grass distribution models may impact predictions of future distributions, as C_3_ grasses from certain clades have less competitive disadvantage compared to C_4_ grasses in a warmer world.

Other environmental changes that can impact C_4_ grass distribution include fire and nitrogen deposition. Fire can influence C_4_ coverage and productivity either through creating open canopies for light capture by C_4_ plants or as a characteristic of semi-arid environments that provide a photosynthetic advantage for C_4_^65^. Recent woody plant encroachment suggests fire had a central role^51,66^ in the formation of grasslands and the rise of C_4_ dominant grasslands in late Neogene^67^ and late Miocene^68^. For recent decades, since globally the trend of fire occurrence is still very uncertain with strong regional variations in the trend^69^, we were unable to quantify its impact on C_4_ grass distribution. Neither the crossover-temperature C_4_ model, nor the optimality model we used, incorporates the role of fire in C_4_ dynamics at present. However, since our approach used annual grassland fractional maps based on satellite remote sensing, the impacts of fire at the annual scale were implicitly considered. Some DGVMs have incorporated fire-relevant processes, however, we found they estimated lower C_4_ grass abundance (i.e., 9.6 ± 6.7%) than those models that do not include fires (i.e.,11.8 ± 3.2%). Additionally, since C_4_ plants have higher photosynthetic nitrogen use efficiency than C_3_^70^, anthropogenic nitrogen deposition^71^ might have impacted the relative advantage of C_4_ to C_3_ photosynthesis. Previous studies have suggested C_4_ plants tend to have a higher photosynthetic rate than C_3_ across a spectrum of nitrogen supply - meaning C_4_ plants have photosynthetic advantage on infertile soils and the advantage will be enhanced by increased nitrogen availability, which can be used to increase C_4_ leaf area^72^. In this study, we used a data-driven product of leaf nitrogen content and remote sensing leaf area index to simulate C_4_ grass distribution (see Methods), which may have implicitly accounted for the effect of nitrogen deposition or limitation on C_4_ photosynthesis.

In this study, we used LUHv2—the primary dataset used in current global carbon cycle modeling and climate forecasting^36,41^—to examine the changes in C_4_ cropland and reported an increase in C_4_ cropland area. However, we note a key source of uncertainty in this dataset: the historical simulation of C_4_ crop distribution used a constant fraction of C_4_ crop cover for each global grid cell, based only on observations circa 2000^41^. Therefore, the increase in C_4_ crop area in LUHv2 could just reflect an increase in all croplands rather than real C_4_ expansion. To reduce the uncertainty caused by this issue, we used another cropland dataset^39^, which dynamically simulated the area of 17 major crop types (including main C_4_ crops maize, millet and sorghum) based on annual FAO census of crop harvested areas. This analysis confirmed our results regarding the increase in C_4_ cropland mainly due to the expansion of maize (Fig. 3f), with a similar spatial pattern reported (Fig. S9). However, the sum of the three main C_4_ species only increased by 0.1%, relatively lower than what we see from the LUHv2 dataset (i.e., 0.4%). We can conclude there was an increase in C_4_ cropland, though the magnitude of increase should be subject to further examinations.

In conclusion, we used a combination of plant photosynthetic pathway records, remote sensing, and an optimality-based photosynthesis model to estimate the global C_4_ coverage and the magnitudes of C_4_ photosynthesis and their variations over the past two decades. We infer that C_4_ vegetation covered on average 17.5% of the global land surface over the period from 2001 to 2019, while C_4_ grass cover decreased due to elevated CO_2_ and C_4_ crop cover increased because of corn (maize) expansion. We predict that C_4_ photosynthesis accounted for 19.5% of the global total photosynthesis, with an increased contribution from C_4_ crops and a decrease from C_4_ natural grasses during this period. Our study offers an updated and more observationally constrained estimate of C_4_ vegetation distribution and photosynthesis, thereby improving our understanding of potential future C_4_ changes and enhancing the quantification of the global carbon budget.

## Methods

### The overarching framework

The distribution of C_4_ vegetation overwhelmingly consists of C_4_ natural grasses and C_4_ crops. To estimate the C_4_ natural grass distribution, we first used an optimality photosynthesis model^44^ to simulate the optimal photosynthetic assimilation rates of C_4_ and C_3_ plants (noted as AC_3_ and AC_4_, respectively) using 0.5 × 0.5 degree gridded historical climate (i.e., CRU-JRA2020), soil^73^ and leaf nitrogen content^74^. We calculated the ratio of AC_4_ to AC_3_, and established a statistically significant (*p* < 0.01) relationship between AC_4_/AC_3_ and the observed C_4_ coverage from multiple databases—the TRY database^45^ and the DG dataset^25^ based on an assumption that larger AC_4_/AC_3_ indicates higher C_4_ grass coverage (% of grassland covered by C_4_ grasses). Using the AC_4_/AC_3_ - C_4_ coverage relationship, we estimated the C_4_ grass coverage (a.k.a. potential C_4_ abundance when grasses cover 100% of the land surface) from estimated AC_4_/AC_3_ for the globe. We lastly overlaid the C_4_ coverage map to a global map of grassland fraction from remote sensing to acquire actual C_4_ grass abundance (% of the land surface covered by C_4_). The workflow is presented in Fig. S1. Meanwhile, for C_4_ crop distribution, we directly used the estimates from the LUHv2-2019 dataset, in which C_4_ crop distribution is estimated from FAO survey and satellite remote sensing.

### Processing observational C_4_ records

We acquired 61,588 georeferenced records of photosynthetic pathways from the TRY database (last accessed 2022 June), among them, there were 2269 records of C_4_. The time range of the record covers  roughly the past 50 years. We first removed the woody species from the records, based on species names and an index table from the TRY database (https://www.try-db.org/TryWeb/Data.php#3), as our study aimed to examine C_4_ grass distribution and the global cover of C_4_ forests is not extensive^47^. After the step, we kept 13,919 records for non-woody species, among which 1963 were C_4_. We further removed 82 records that belong to major C_4_ crops (i.e., maize, sugarcane, millet, and sorghum) and kept 1881 records. We then aggregate these records to 10 × 10 degree cells, in each cell we calculate the species richness of C_4_ (i.e., number of C_4_ species/total number of herbaceous species, the numbers were derived from the available records in the TRY database). The gridded values of C_4_ species richness would be further used to constrain the optimality model to estimate global C_4_ grassland coverage. Here we use the large-size grid cell to make sure there were enough samples in each cell to acquire a meaningful estimate of C_4_ abundance – in this analysis, each cell should have at least 50 species (i.e., C_3_ and C_4_ in total). We used 1619 (out of 1881) C_4_ species records in this aggregation step, and obtained 23 10 × 10 degree cells for the analysis (Fig. S2).

Note here the C_4_ abundance from the TRY database was species richness, not equal to the area abundance that is often used in DGVMs. To acquire C_4_ grass coverage (% of grassland covered by C_4_) from C_4_ species richness (% of grass species that is C_4_), we used an open dataset from the global nutrient network (NutNet) that has paired C_4_ species richness and C_4_ area abundance (% of the land surface covered by C_4_) to infer their relationship^46^ (Fig. S3). The dataset includes species-specific coverage records as well as the grass species richness data collected in 25 m^2^ plots across 34 sites. Each site has between 1 and 6 control plots. We only used the data from the control plots, excluding plots that underwent nutrient addition treatments. To avoid the uneven distribution of data samples, we grouped the paired observations by their C_4_ species richness, and for each species richness we get a mean C_4_ area abundance and the standard deviation of the C_4_ grass coverage. We then conducted 1000 linear fittings (i.e., with an intercept of 0, since C_4_ grass coverage should be 0 when C_4_ species abundance is 0), and for each fitting we used randomly sampled C_4_ grass coverage values (i.e., based on mean and the standard deviation) value against C_4_ species richness values. The slopes of the linear regressions represented a conversion factor between C_4_ species richness and C_4_ grass coverage (Fig. S3).

In addition to the TRY database that has a global representation, we also used a gridded C_4_ grass coverage data compiled for the contiguous United States (denoted as the DG dataset)^23^. The DG dataset provides C_4_ grass coverage (% of grassland) aggregated at a 100 km resolution grid, which was sampled from roughly 40,000 plots over the past 40 years. Please note that the DG dataset only surveyed C_4_ grass species. We used the DG dataset and the TRY database to establish the relationship between AC_4_/AC_3_ and C_4_ grass coverage (Fig. 1b).

### Processing cropland and land use data

We used a gridded C_4_ crop distribution from the LUHv2 dataset (version: LUHv2-GCB2019)^36^. It was estimated based on the FAO census and national reporting of >170 major crop types (including the main C_4_ types), supplemented by the total cropland area collated by HYDE3.2^40^ which came from FAO census and remote sensing products. The C_4_ crop fraction of each grid cell was only acquired based on observations circa 2000^37^ and the fraction was kept constant over the study period. The most recent versions of the LUHv2 dataset have been used in CMIP6 for the IPCC AR6 report and the global carbon budget. Since the LUHv2 dataset did not contain an estimate of uncertainty, we relied on an independent study that compared four different land use products (including LUHv2) and reported the uncertainty of cropland estimation between products was about 10%^75^. We thus used 10% to represent the uncertainty range of the C_4_ cropland area.

In addition to LUHv2, we used another open dataset reporting the area of 17 main crop types from 1961 to 2014^39^. Unlike the LUHv2 dataset which almost exclusively relied on observations circa 2000 to quantify the C_4_ crop fraction, this other dataset used annual FAO census records for crop area fraction estimation, including those of the three primary C_4_ crops: maize, millet, and sorghum. We used this dataset to examine the changes in global C_4_ croplands and compare to the values obtained from the LUHv2 dataset.

### The optimality model for C_4_ and C_3_ photosynthesis

We used optimal C_3_ and C_4_ photosynthesis models to simulate optimal C_3_ and C_4_ photosynthesis^44^. The soil-plant-air water continuum was incorporated in C_3_ photosynthesis models^76^ and C_4_ photosynthesis models^77^ to examine interactions of CO_2_, water availability, light and temperature. The model considered optimal stomatal resistance and leaf/fine-root allocation to maximize the carbon gain regarding water loss, and successfully predicted the ancient distribution of C_4_ species in Oligocene and Miocene^44^.

In the current study, we improved the modeling processes through the following aspects. (1) We used different parameters for C_3_ and C_4_ species specifically to better represent the diversity of C_3_ and C_4_ species variability (Table S2 in the Supplementary Note). (2) We considered the effects of nitrogen availability and optimal nitrogen allocation between C_3_ and C_4_ species. Specifically, we adjusted the maximum carboxylation rate (*V*_cmax_) and maximum electron transport rate (*J*_max_) values using optimal *J*_max_/*V*_cmax_ ratio (i.e., 2.1 for C_3_ and 5.0 for C_4_, which were supported by both measurements and theoretical modeling)^78,79^ and available leaf nitrogen content for C_3_ and C_4_ respectively. (3) Since a large majority of C_4_ species are herbaceous, when we modeled closed canopy biomes (e.g., those pixels dominated by tree and shrubs), we used estimates of understory photosynthetic active radiation (PAR) to model the relative advantage of the herbaceous species. A full model description and the parameterization is in the Supplementary Note.

Using the models, we were able to calculate the optimal assimilation rates for C_3_ and C_4_ (i.e., AC_3_ and AC_4_) over the globe at the 0.5-degree resolution (i.e., dependent on the spatial resolution of climate input), where the relative advantage of C_4_ to C_3_ is defined as AC_4_/AC_3_. The simulation was conducted at an annual time step and there was no need for model initialization. When establishing the relationship between AC_4_/AC_3_ and C_4_ grass coverages (Fig. 1b) from the DG and TRY datasets, we aggregated the simulations from 0.5-degree to 1-degree (approximately 100 km at the equator) and 10-degree. As the relationships derived from both 10-degree (i.e., TRY) and 1-degree (i.e., DG) data were similar, we assumed that the relationship is scale-independent. Consequently, we applied it to 0.5-degree estimates of AC_4_/AC_3_ to infer global C_4_ grass coverage. We also assumed that the relationship between AC_4_/AC_3_ and C_4_ grass coverage was time-invariant.

### Running the optimality model

We used annual growing season average soil water potential, vapor pressure deficit (VPD), 2 m daytime air temperature (T_air_), photosynthetic active radiation (PAR) and leaf nitrogen content as inputs for the optimality photosynthesis model. The growing season was defined using the MODIS phenology product (MCD12Q2)^80^.

T_air_ was acquired from the CRU-JRA2020 dataset. VPD was estimated using the specific humidity and air temperature from CRU-JRA2020. Soil water potential was estimated from soil texture properties from soil grids and global soil water content datasets, using the Clapp & Hornberger equation^81^. The global soil water content datasets came from GLEAM v3^82^. To avoid extreme low soil water potential that does not allow plant growth in the optimality model, we set the minimal soil water potential to −3 MPa. The leaf nitrogen content was acquired from a machine learning upscaled leaf traits product^74^. PAR was also acquired from the CRU-JRA2020 dataset, which is a reanalysis from CRU^83^ and JRA^84^. We directly used PAR for ‘open’ ecosystems (i.e., grasslands, savannas), however, for dense forests and shrublands we used understory PAR (i.e., as C_4_ grasses often exist in understories), which was derived from PAR and multi-year average MODIS LAI^85^ (i.e., assume they are overstory LAI) following a radiation gradient mandated by the Beer’s Law.

We ran the photosynthetic optimality models multiple times in the process. We first modeled the growing season AC_4_/AC_3_ in the study period using the climatology of the variables mentioned from 2001 to 2019. To model the growing season AC_4_/AC_3_ from 2001 to 2019, for each year we used the 20-year climatology (i.e., 20 years before the target year) of the driving variables. In addition, we also conducted simulation for four scenarios, in which we replaced the climate input for 2001 simulation with the CO_2_, T_air_, VPD and soil moisture from 2019, respectively. Then we used AC_4_/AC_3_ to estimate the C_4_ grass distribution for each year or for each scenario. By calculating the difference between the C_4_ grass distribution of four scenarios and the C_4_ grass distribution in 2001, we quantified the contribution of CO_2_, T_air_, VPD and soil moisture to the changes in C_4_ grass distribution.

### Remote sensing estimates of global grassland fraction

Multiple remote sensing products provide information on grassland distributions. Some directly provide continuous fraction values (i.e., GLC^86^ at 100 meter and Dynamic World^87^ at 10 meter) and some provide categoric information on grassland and savannas (i.e., MODIS^88^ at 500 meter and ESA-CCI^89^ and 300 meter). For the former, we can directly calculate the grassland fraction value at 0.5-degree resolution; for the latter, we assign the grassland/savanna type pixel to 100% and others to 0% grassland, and then obtain the mean value for each 0.5 grid cell. We found that those four estimates of grassland fraction vary considerably (Fig. S4). Based on a visual comparison of the four estimates (i.e., GLC, Dynamic World, MODIS and ESA-CCI) against vegetation map estimates^90^, we found Dynamic World and ESA-CCI substantially underestimate grassland fraction. We therefore used only MODIS and GLC estimates of grassland fractions in our study.

The MODIS grassland fraction product is available from 2001 to 2019. The GLC product is only available from 2015 to 2019. To extend the GLC product back to 2001, we employed a random forest approach to estimate GLC estimates based on surface reflectance, climate, and soil type and extrapolate it to 2001 (i.e., the training accuracy is 99% and the validation accuracy is 95%). We used the average of MODIS and GLC estimates to represent the grassland fraction. To quantify the uncertainty of the approach, for each pixel we bootstrapped 1000 times between the MODIS estimate and the GLC estimate, and use the one standard deviation of these 1000 values to represent the uncertainty in grassland fraction.

### Dynamic Global Vegetation Models (DGVMs)

We used 11 DGVMs participating in the global carbon project^91^(Table S1) in our study. Though all of the 11 DGVMs provided simulations for C_4_ natural grasses, only 7 of them have simulations for C_4_ crops (Table S1). We established an emergent constraint between C_4_ area and C_4_ photosynthesis contribution using the estimates from the model ensemble. We used the S3 scenario (i.e., considering elevated CO_2_, climate change and land use change) of model simulations in our analysis.

### Emergent constraint approach

The emergent constraint technique is widely used in climate and modeling communities to infer unobserved quantities of interest in land surface processes^92,93^. The underlying assumption is that although there is a large spread in the model estimates of an observed variable X and an unobserved variable Y across models, the relationship linking the two is tightly constrained across models. Based on the strong and robust relationship across models between X and Y, observations of X can be used to generate a constraint on unobserved Y. This approach has been termed ‘emergent’ because the functional relationship cannot be diagnosed from a single model, but rather emerges from the spread of the model estimates. The emergent constraint identified in this study links the contribution of C_4_ grasses/crops to total GPP to the percentages of area covered by C_4_ grasses/crops as estimated from the ensemble of DGVM simulations.

### Reporting summary

Further information on research design is available in the Nature Portfolio Reporting Summary linked to this article.

### Supplementary information

Supplementary Information

Peer Review File

Reporting Summary

## Data Availability

The global C_4_ vegetation distribution map is available at https://zenodo.org/records/10516423. The CS C_4_ map was acquired from https://daac.ornl.gov/cgi-bin/dsviewer.pl?ds_id=932. The CRU TS4.02 climate data is available at https://crudata.uea.ac.uk/cru/data/hrg/, the soil moisture data can be downloaded from https://www.gleam.eu/#datasets. The global dataset of leaf photosynthetic pathway was acquired from the TRY database https://www.try-db.org/TryWeb/Home.php, by selecting those records with the field “photosynthesis pathway (traitID: 22)”. The DG dataset was obtained from the supporting information of https://onlinelibrary.wiley.com/doi/10.1111/jbi.13061. The subset of the observations from the nutrient network (NutNet) are accessible at https://portal.edirepository.org/nis/mapbrowse?packageid=edi.1037.2.

## Code availability

The code for analysis is available at https://github.com/lxzswr/C4distribution/ and https://zenodo.org/records/10516423. The code of optimality photosynthesis model is available at https://github.com/zhouhaoran06/C3C4OptPhotosynthesis-. All maps in the study were generated with the assistance of the ‘M_Map’ package in Matlab, the instruction of which is available at https://www.eoas.ubc.ca/~rich/map.html.
