# Supplementary material for: Mapping the global distribution of C4 vegetation using observations and optimality theory
Source: Nat Commun. 2024 Feb 9;15:1219. doi: 10.1038/s41467-024-45606-3 (PMC10858286; doi:10.1038/s41467-024-45606-3)
Supplement: Supplementary file 1 — Supplementary Information [file 41467_2024_45606_MOESM1_ESM.pdf]

Supplementary Information

Mapping the global distribution of C_4 vegetation using observations and optimality theory

Supplementary Figures

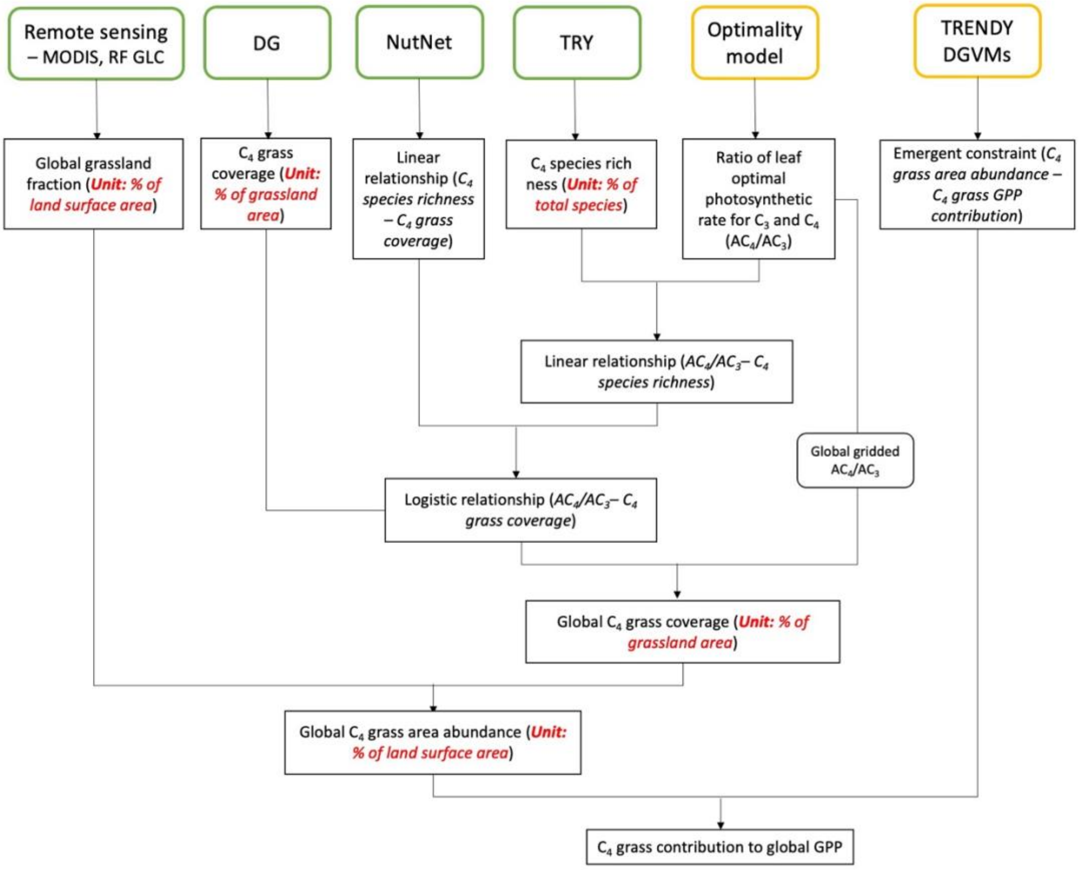

Figure S1. The workflow of estimating C_4 natural grass distribution and GPP from observations and the optimality model. Green boxes indicate observations, yellow boxes indicate models. Units and relationships between variables are highlighted in italic.

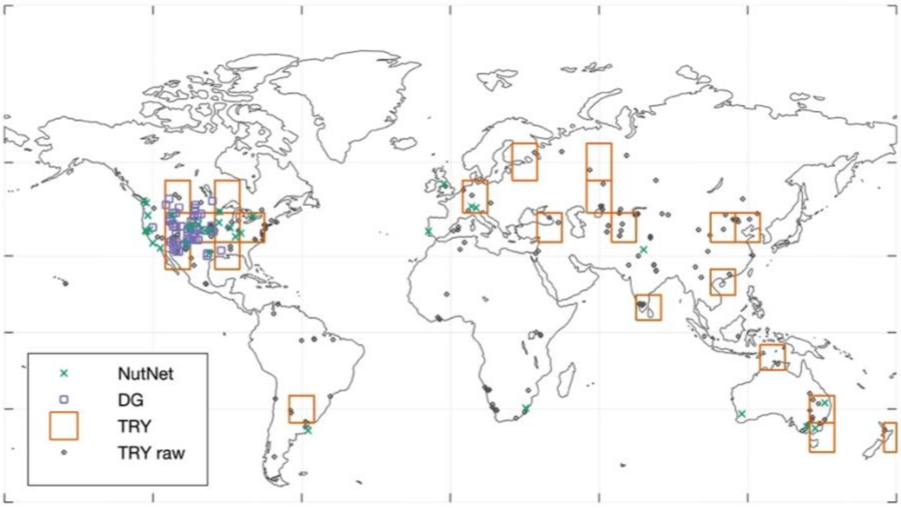

Figure S2. The spatial distribution of C_4 plant observations used in the study. "TRY raw" means the locations where there are C_4 observations in the TRY database, "TRY" is the aggregated TRY raw observations at 10-degree spatial resolution (i.e., each aggregated grid cell for TRY has more than 50 species recorded, including C_3 and C_4), "DG" means the C_4 observations compiled for North America at 100 km spatial resolution, "NutNet" indicates some sites from the Nutrient Network where we obtained the observations of grass species richness and species coverage.

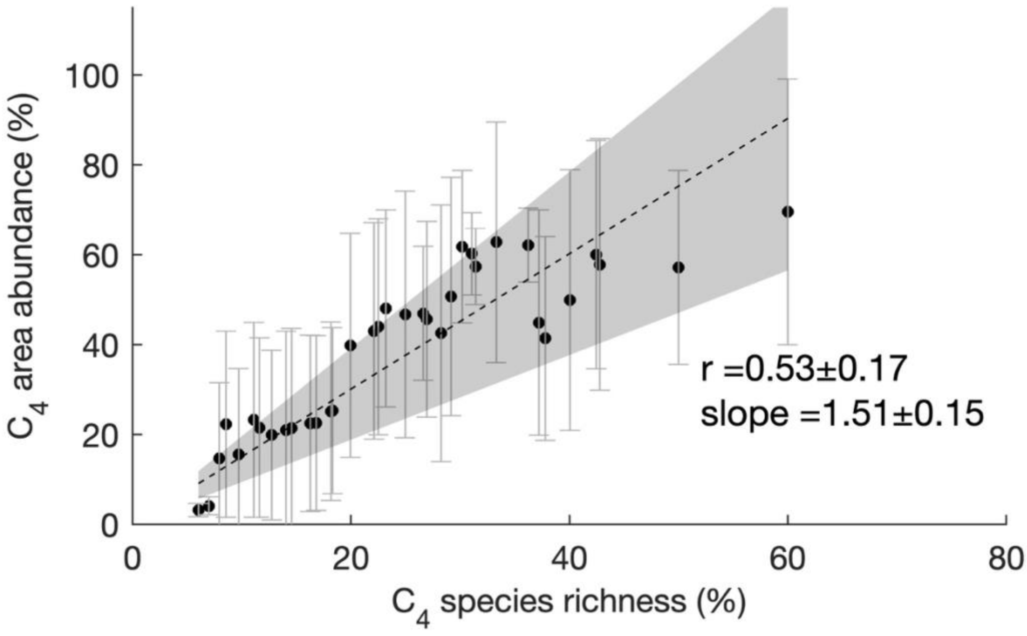

Figure S3. The observed relationship between C_4 species abundance and C_4 area abundance. Error bars indicate one standard deviation of C_4 area abundance observations under each C_4 species abundance. The shaded area indicates one standard deviation of the slope values of 1000 linear regressions between C_4 species and area abundances. For each regression we fitted randomly sampled C_4 area abundance against the C_4 species abundance. The C_4 area and species abundance observations were acquired from the global nutrient network.

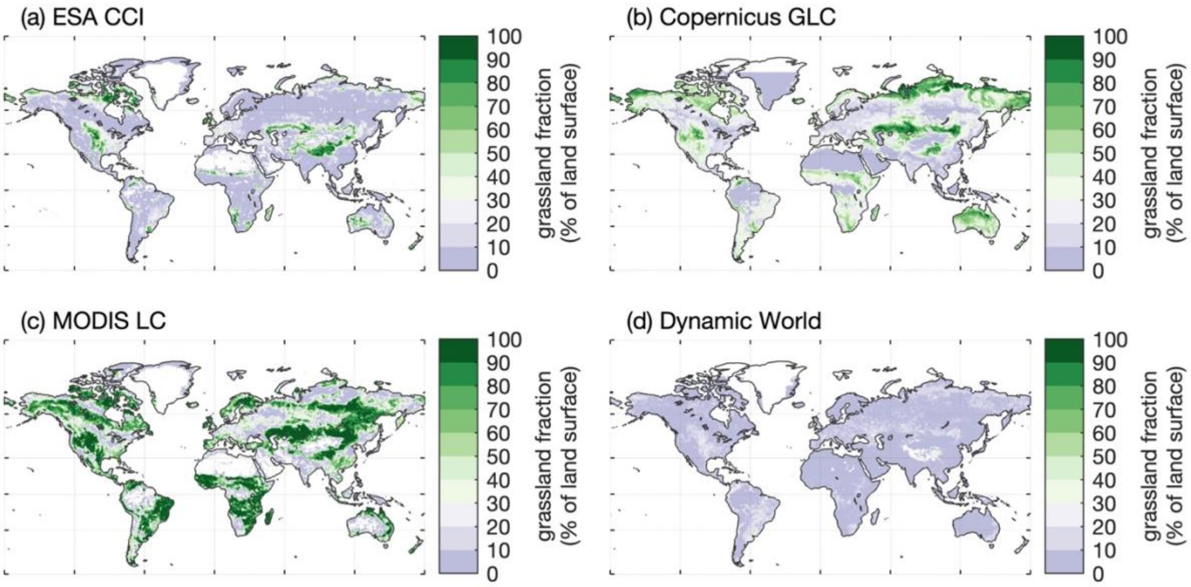

Figure S4. The global grassland fraction provided by four mainstream remote sensing products. (a) ESA-CCI; (b) Copernicus GLC; (c) MODIS Land Cover and (d) Dynamic World.

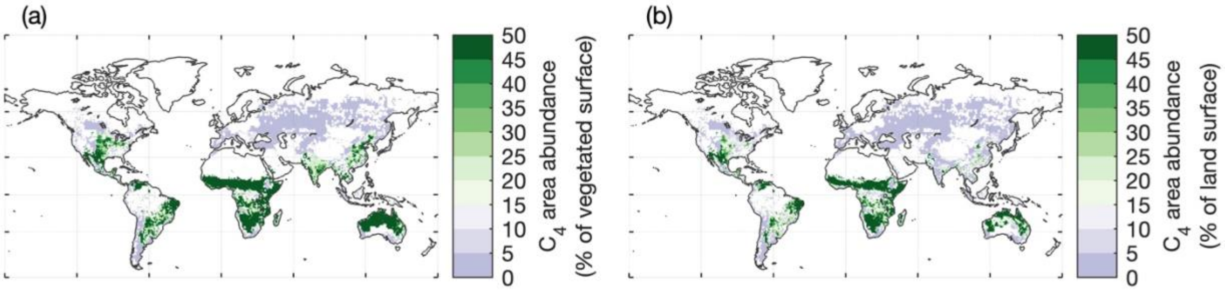

Figure S5. The C_4 vegetation distribution estimated by the crossover temperature hypothesis. (a) C_4 area abundance in % of the vegetated surface; (b) C_4 area abundance in % of the land surface.

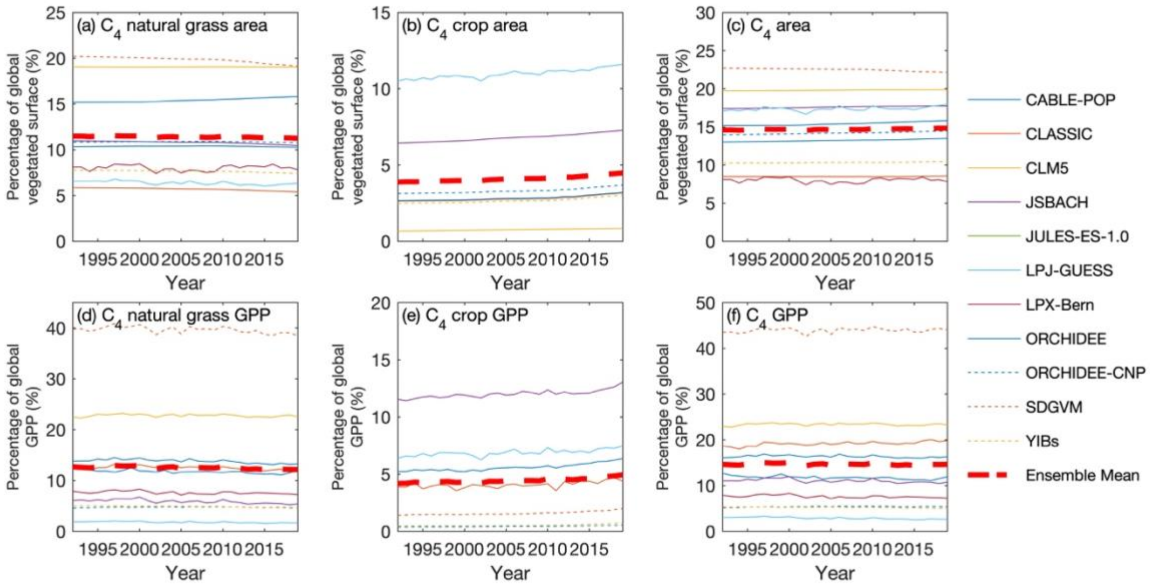

Figure S6. The changes in C₄ area abundance and photosynthesis from DGVMs. (a, b, c) The changes in the area of C_4 natural grasses, C_4 crops and total C_4, in terms of percentage of global vegetated surface; (d, e, f) The changes in the photosynthesis (i.e., gross primary productivity) of C_4 natural grasses, C_4 crops and total C_4 photosynthesis, in the unit of percentage of global photosynthesis.

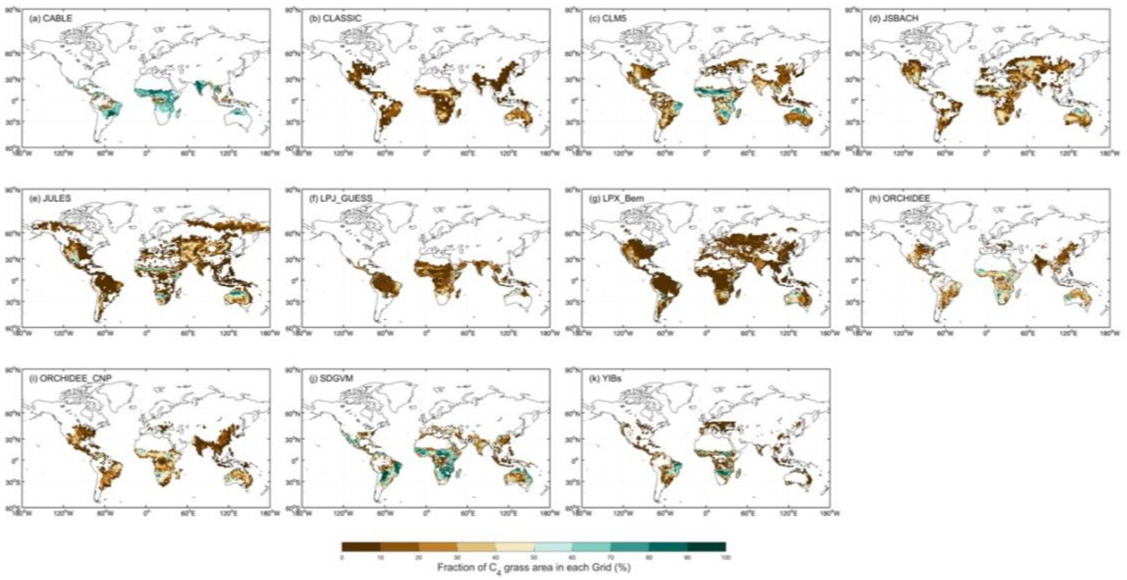

Figure S7. The spatial distribution of C_4 grass estimated by 11 Dynamic Global Vegetation Models (DGVMs) in TRENDY v9 project.

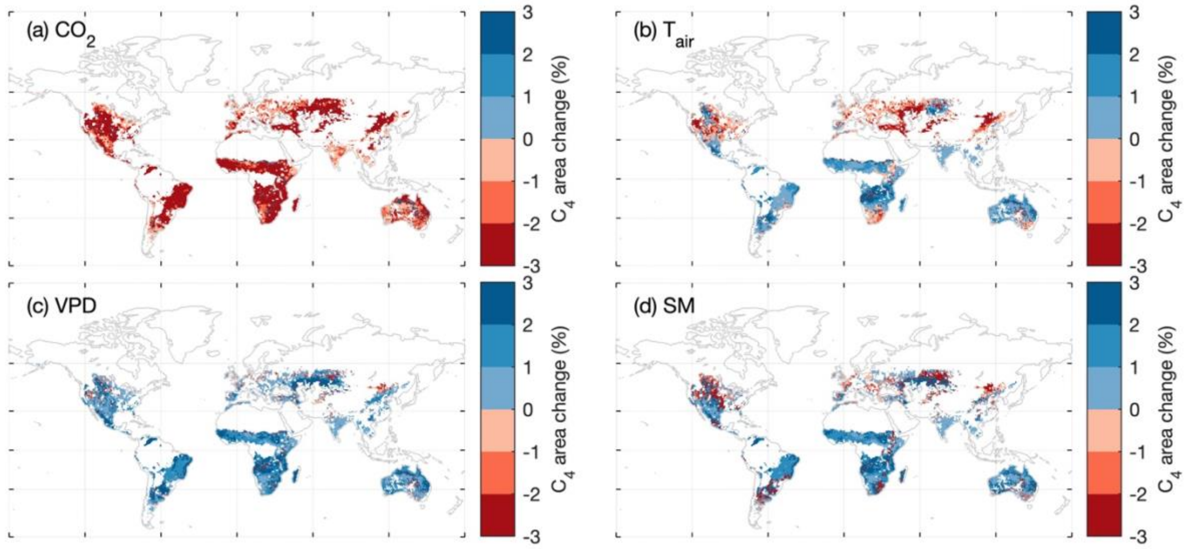

Figure S8. The impacts of climate drivers on C_4 natural grasslands change from 2001 to 2019. (a) CO_2, (b) T_{air}, (c) VPD and (d) soil moisture.

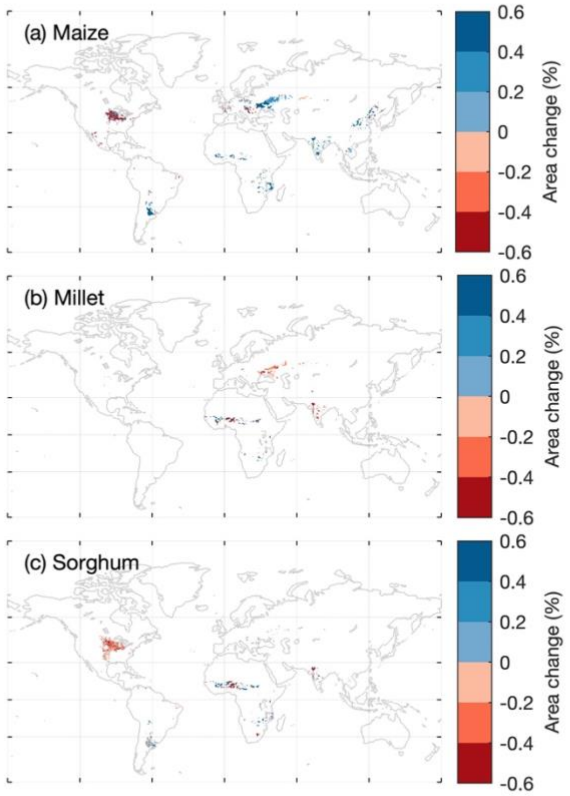

Figure S9. The changes in the areas of three major C_4 crops from 2001 to 2014. (a) Maize, (b) Millet and (c) Sorghum.

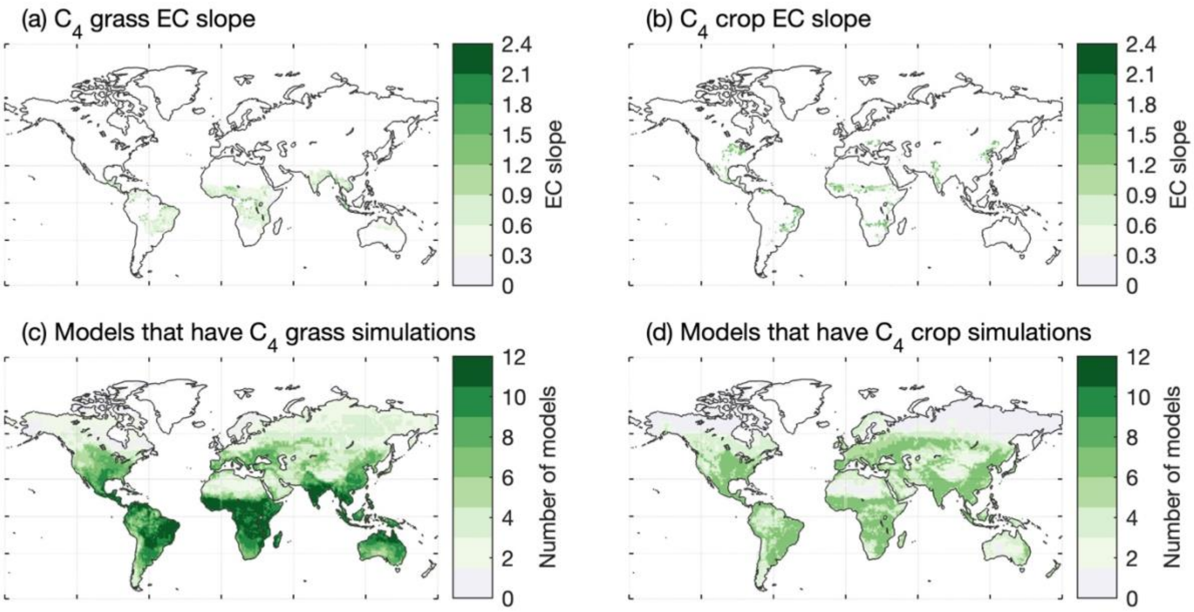

Figure S10. The emergent constraint at the pixel level. The slopes of the emergent constraint for (a) C_4 grass and (b) C_4 crop. We only show the slopes where the relationship between C_4 area abundance and C_4 GPP percentage is significant ($p<0.05$). The numbers of DGVMs that have simulations for (c) C_4 grass and (d) C_4 crop.

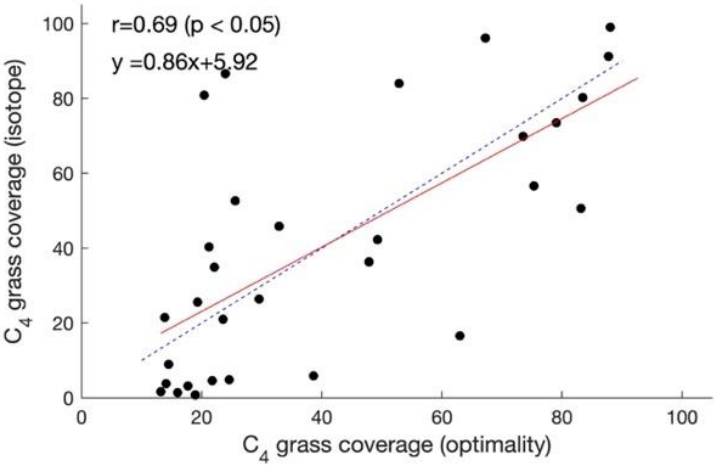

Figure S11. The correlation between Australian C_4 grass coverage estimated by the optimality model used in our study (on the x-axis) and the C_4 grass coverage estimated by isotope measurements and remote sensing in Munroe et al. 2022 (on the y-axis). Each data point represents one of the continental Australian bioregions defined by the Interim Biogeographic Regionalisation for Australia version 7 (IBRA 7.0). It's important to note that the actual number of data points is lower than the total number of Australian bioregions due to some bioregions lacking C_4 estimates from our 0.5-degree C_4 map and others reporting C_4 grass coverage (isotope) exceeding 100%. The formula used for calculating C_4 grass

coverage (isotope) is 'Min_Proportional_C_4_Cover' divided by 'Mean_%_herbaceous_cover', with the values obtained from the supplementary table of Munroe et al. 2022.

Supplementary Tables

Table S1. The Dynamic Global Vegetation Models (DGVMs) from TRENDY v9, and the availability of their simulations of C_4 area and gross primary productivity (GPP).

Model	Total number of Plant Functional Types (PFTs)	C_4 natural grass	C_4 crop	Note
CABLE-POP	10	Yes	No	
CLASS	10	Yes	Yes	
CLM5	78	Yes	Yes, but most crop types do not have GPP values provided	In model description PFT-14 is regarded as C_4 grass, but the actual output of C_4 grass is PFT-15.<br><br>C_4 crop includes Corn (Maize), Millet, Sorghum and Sugarcane.
JSBACH	14	Yes	Yes	C_4 crop includes C_4 crop and pasture
JULES-ES-1.0	12	Yes	Yes	C_4 crop includes C_4 crop and pasture

LPJ-GUESS	26	Yes	Yes	C_4 crop includes C_4 annual, C_4 perennial, C_4 annual irrigated, C_4 perennial irrigated, C_4 cover crop grass and C_4 pasture
LPX-Bern	20	Yes	Yes	C_4 crop includes C_4 crop and pasture
ORCHIDEE	15	Yes	Yes	
ORCHIDEE-CNP	15	Yes	Yes, but not used, as GPP value provided is close to 0	
SDGVM	10	Yes	Yes	
YIBs	8	Yes	Yes, but not used as GPP value provided is close to 0	

Supplementary Notes

Detailed description of the optimality C_3 and C_4 photosynthesis model

The model is based on the optimality model of C_3 and C_4 models constructed in Zhou *et al.* (2018)[1]. The model incorporated the soil-plant-air water continuum into traditional C_3 and C_4

photosynthesis models[2,3]and maximized assimilation rate by optimizing stomatal conductance and root/shoot allocation.

C_3 photosynthesis model

Considering the steady state of CO_2 diffusion in mesophyll cells, we get:

$$A_n = \frac{C_a - C_m}{r_s + r_m}, \qquad (1)$$

where A_n is the net assimilation rate, C_a and C_m are the atmospheric and mesophyll CO_2 mixing ratios, and r_s and r_m is the stomatal and mesophyll resistance (the inverse of stomatal or mesophyll conductance). A_n is computed using the FvCB model[2] and is the minimum of two limitation states (eq. (4)): the Rubisco carboxylation (dark reaction) limitation state (A_c) (eq. (2)), low CO_2 and high light intensity cause a saturating supply of substrate (RuBP) for Rubisco, and reaction rate is controlled by the enzyme kinetics of Rubisco; the RuBP regeneration (light reaction) limitation state(A_j) (eq. (3)), when light intensity is low and RuBP availability limits the reaction rate. The assimilation rates are given by:

$$A_c = \frac{V_{cmax}(C_m - \gamma^* O_m)}{C_m + K_c(1 + O_m/K_o)} - R_d \qquad (2)$$

$$A_{j,atp} = \frac{J_{max}(C_m - \gamma^* O_m)}{4.5C_m + 10.5\gamma^* O_m} - R_d \qquad (3)$$

$$A_n = \min\,(A_c, A_j) \qquad (4)$$

where V_{cmax} is maximum velocity of Rubisco carboxylation, J_{max} is maximum rate of electron transport at a specific light intensity, R_d is the mitochondrial respiration rate in the daytime and γ^* is half the reciprocal of Rubisco specificity. O_m is O_2 concentration in the mesophyll cell, which is assumed equal to atmospheric O_2. K_c and K_o are the Michaelis-Menten constants of Rubisco for CO_2 and O_2.

C_4 photosynthesis model

For the C_4 pathway, we consider the steady state mixing ratio of CO_2 in both mesophyll (C_m) and bundle sheath cells (C_{bs}), which gives us two equations:

$$A_g + R_{dbs} = V_p - g_{bs}(C_{bs} - C_m) \qquad (5)$$

$$A_n = \frac{C_a - C_m}{r_s + r_m}, \qquad (6)$$

where g_{bs} is the bundle sheath conductance, V_p is the PEP carboxylation rate, (g_{bs}(C_{bs}-C_m)) represents bundle sheath leakage from bundle sheath cells back to mesophyll and R_{dm} are the

daytime mitochondrial respiration rate in mesophyll cells.

The PEP carboxylation rate V_p is limited by either PEPc carboxylation (eq. (7)), which follows a Michaelis-Menten type or PEP regeneration (eq. (8))

$$V_{pc} = \frac{V_{pmax} C_m}{C_m + K_p} \quad (7)$$

$$V_{pr} = xJ_{max}/3 \ , \quad (8)$$

$$V_p = \min\left(V_{pc}, V_{pr}\right), \quad (9)$$

where V_{pmax} is maximal PEPc carboxylation rate, K_p is the Michaelis-Menten coefficient of PEPc for CO_2 and x is the fraction of total electron transport could be used for the PEP regeneration, which represents the cost of the CCM. The denominator 3 in eq. (8) arises due to the fact that regeneration of 1 molecule of PEP needs 2 additional ATP, which is 3 additional electrons transported. Thus, equations (8) and (10) incorporate the cost of C_4 pathway. A_c and A_j of C_4 are given by

$$A_c = \frac{V_{cmax}(C_{bs} - \gamma^* O_{bs})}{C_{bs} + K_c(1 + O_{bs}/K_o)} - R_d \quad (9)$$

$$A_{j,atp} = \frac{(1-x)J_{max}(C_{bs} - \gamma^* O_{bs})}{4.5C_{bs} + 10.5\gamma^* O_{bs}} - R_d \quad (10)$$

which is obtained by substituting C_m in eq. (2) and (3) with C_{bs}.

Based on equations (7), (8), (9) and (10), A_n of C_4 is limited by four states as follows:

$$A_n = \min\left(A_{cc}, A_{cj}, A_{jc}, A_{jj}\right). \quad (11)$$

Here, A_{cc} is RuBP carboxylation and PEPc carboxylation limited rate; A_{cj} is RuBP carboxylation and PEP regeneration limited rate; A_{cj} is PEP carboxylation and RuBP regeneration limited rate; and A_{jj} is limited by PEP regeneration and RuBP regeneration limited rate.

Hydraulic system

Eq. (12) describes the soil-plant-air continuum[4]. At equilibrium, the rate of water loss through transpiration equals the rate of water absorption by the roots:

$$\frac{EfN}{\rho} = k(1-f)N(\psi_l - \psi_s) \ , \quad (12)$$

where Ψ_s is soil water potential, k is the effective root hydraulic conductivity, N is the total biomass of fine root and leaves, ρ is the leaf mass density (gcm^{-2}) and E is the transpiration rate per leaf area. E could be written as δ/r_s, where δ is the water partial pressure deficit between saturated leaf surface and the atmosphere. Thus, leaf water potential (Ψ_l) is a

function of r_s and leaf/fine-root allocation (f, defined as investment into leaves/total investment in leaves and fine root).

$$\psi_l = \psi_s - \frac{1.6 f \delta}{\rho k r_s (1-f)} \quad (13)$$

Inhibition of photosynthesis by water stress

Reduced leaf water potential inhibits photosynthesis[5,6,7]. We model this cost of transpiration as Weibull-type vulnerability curves relating leaf Ψ_l and photosynthetic parameters[8]:

$$V_{cmax}(\psi_l) = V_{cmax} e^{-(\frac{-\psi_l}{d_v})^{b_v}} \quad (14)$$

$$J_{max}(\psi_l) = J_{max} e^{-(\frac{-\psi_l}{d_j})^{b_j}} \quad (15)$$

$$V_{pmax}(\psi_l) = V_{pmax} e^{-(\frac{-\psi_l}{d_p})^{b_p}}, \quad (16)$$

where b and d are curve fitting parameters. Since Ψ_l is a function of r_s and f, all those parameters are functions of r_s and f.

Optimal stomatal resistance and optimal allocation of energy between leaves and fine roots

We assume that the plant adjusts the r_s and f to optimize the total carbon gain by

$$A_{total} = \frac{f N A_n}{\rho}, \quad (17)$$

where ρ is the leaf mass density (g cm^{-2}). As a simplifying assumption, we assume N and ρ are fixed (similar to Givnish, 1986[4]). Effectively, we consider the optimization problem faced by the plant in a given instance during its growth, where its size (of which N is a proxy) can be regarded as a constant. Clearly, during plant growth, the assimilate will be turned into plant biomass, but the instantaneous optimization problem will still yield the optimal growth path, as it maximized the growth rate at any given time. Finally, we regard ρ as a species-specific trait that changes at a slower time-scale than r_s and f. The first order optimality conditions for r_s and f are given by[4]:

$$\frac{\partial (f A_n)}{\partial r_s} = f \frac{\partial A_n}{\partial r_s} = 0 \quad (18)$$

$$\frac{\partial (f A_n)}{\partial f} = A_n + f \frac{\partial A_n}{\partial f} = 0\,. \quad (19)$$

We checked the second order derivative to ensure that the numerical solutions to the first order conditions were maxima.

4 Temperature and light dependence of parameters

In the models, K_c, K_o, V_{cmax}, V_{cmax}, J_{max}, V_{pmax}, K_p, γ^* and g_m are temperature dependent. The temperature dependence equations for K_c, K_o, γ^*, and K_p follow the Arrhenius function:

$$\mathrm{R} = \mathrm{R}(25)e^{E_a\frac{T_k-298.15}{298.15RT_k}}, \qquad (20)$$

where $R(25)$ is the value of parameter at 25 °C, ΔH_a represents enthalpies of activation in kJ mol^{-1}, R is the molar gas constant of 0.008314 kJ k^{-1}mol^{-1}, T_k is the leaf temperature in K. The temperature dependence equation for g_{bs}, g_m, J_{max} and V_{pmax} follow the modified Arrhenius function:

$$\mathrm{R} = \mathrm{R}(25)e^{E_a\frac{T_k-298.15}{298.15RT_k}} \times \frac{1+e^{\frac{298.15\times\Delta S-H_d}{298.15R}}}{1+e^{\frac{T_k\times\Delta S-H_d}{T_kR}}}, \ (21)$$

Where ΔH_d is a term of deactivation in kJ mol^{-1} and ΔS is a term of entropy in kJ mol^{-1} k^{-1}.

When the light intensity varies, the following function is used to adjust the electron transport rate[9]:

$$J = \frac{I_2+J_{max}-\sqrt{(I_2+J_{max})^2-4\Theta I_2J_{max}}}{2\Theta}. \qquad (22)$$

Optimal photosynthesis parameters (J_{max}, V_{cmax} and V_{pmax}) for C_3 and C_4

Nitrogen allocation between RuBP carboxylation, RuBP regeneration in C_4 grasses affect competitive advantage over C_3 grasses. Despite great variation in V_{cmax} and J_{max} based on the total leaf nitrogen content within C_3 plants, Wullschleger (1993)[10] found a mean of J_{max}/V_{cmax} =2.1 across 109 C_3 species, which is consistent with the modeled optimal results. Then, we used the modeled optimal value of J_{max}/V_{cmax} =5 and V_{pmax}/V_{cmax} =2 for C_4[11]. In determining the values of J_{max} and V_{cmax}, we used the stoichiometry in Zhou et al. (2023)[11]. Thus, for nitrogen availability in each pixel, we calculated corresponding J_{max} and V_{cmax} for C_3 and C_4.

Table S2. Input parameters used for modeling optimal J_{max}/V_{cmax} and V_{pmax}/V_{cmax} for C_3 and C_4.

Parameter	Value		unit	Definition
	C3	C4		

$K_c(25)$	302[12]	450.7[3,13,14,15,16,17,18,19,20]	μbar	Michaelis-Menten constant of Rubisco activity for CO_2 at 25°C
$K_o(25)$	256[12]	354.65[3,13,14,15,16,20]	mbar	Michaelis-Menten constants of Rubisco activity for O_2
γ^* (25)	0.000171[4]	0.000244[3,13,15,16,17,18,19,20]		Half of the reciprocal of Rubisco specificity
$g_m(25)$	3[21]	7.5[20,21,22]	μmol m^{-2} $s^{-1}Pa^{-1}$	mesophyll conductance at 25°C
$K_p(25)$		84.65[3,13,14,23]	μbar	Michaelis-Menten constants of PEP carboxylation for CO_2
ΔH_a for V_{cmax}	65.33[24]	51.89[13,19,23]	kJ mol^{-1}	energy of activation for temperature dependence for V_{cmax}
ΔH_a for J_{max}	43.9[25]	69.25[14,26]	kJ mol^{-1}	energy of activation for temperature dependence for J_{max}
ΔH_a for V_{pmax}		65.69[13,14,23,26]	kJ mol^{-1}	energy of activation for temperature dependence for V_{pmax}
ΔH_a for K_o	36.38[24]	12.8[13,23]	kJ mol^{-1}	energy of activation for temperature dependence for K_o
ΔH_a for K_c	79.43[24]	36.5[13,17,19]	kJ mol^{-1}	energy of activation for temperature dependence for K_c
ΔH_a for γ^*	37.83[24]	24.82[13,17,19,23]	kJ mol^{-1}	energy of activation for temperature dependence for γ^*
ΔH_a for K_p		52.2[13,23]	kJ mol^{-1}	energy of activation for temperature dependence for K_p
ΔH_a for g_m	49.6[27]	46.533[20]	kJ mol^{-1}	energy of activation for temperature dependence for g_m
ΔH_a for g_{bs}		116.77[23]	kJ mol^{-1}	energy of activation for temperature dependence for g_{bs}
ΔH_d for J_{max}	200[25]	188.502[14,26]	kJ mol^{-1}	energy of activation for temperature dependence for J_{max}
ΔH_d for V_{pmax}		147.694[13,14,23,26]	kJ mol^{-1}	energy of activation for temperature dependence for V_{pmax}
ΔH_d for g_m	437.4[27]	366.8[20]	kJ mol^{-1}	energy of activation for temperature dependence for g_m
ΔH_d for g_{bs}		264.6[23]	kJ mol^{-1}	energy of activation for temperature dependence for g_{bs}
ΔS for J_{max}	0.65[25]	0.609[14,26]	kJ K^{-1} mol^{-1}	entropy for temperature dependence for J_{max}
ΔS for V_{pmax}		0.47[13,14,23,26]	kJ K^{-1} mol^{-1}	entropy for temperature dependence for V_{pmax}
ΔS for g_m	1.4[27]	1.2[20]	kJ K^{-1} mol^{-1}	entropy for temperature dependence for g_m
ΔS for g_{bs}		0.86[23]	kJ K^{-1} mol^{-1}	entropy for temperature dependence for g_{bs}
ρ	18.3[4]	38.4[28,29,30]	g m^{-2}	leaf mass density
b_v	3.8[8]	3.8[8]		Weibull-type vulnerability curve for V_{cmax}
d_v	2[8]	2[8]	MPa	Weibull-type vulnerability curve for V_{cmax}
b_j	3[8]	3[8]		Weibull-type vulnerability curve for J_{max}
d_j	2.5[8]	2.5[8]	MPa	Weibull-type vulnerability curve for J_{max}
K_{plant}	0.001044[4]	0.002088[4,21,31]	gH_2O g^{-1} MPa^{-1} s^{-1}	hydraulic conductivity
g_{bs}		0.02946[3,14,23,32]	μmol m^{-2} $s^{-1}Pa^{-1}$	bundle sheath conductance for CO_2

Supplementary References

1. Zhou, H., Helliker, B.R., Huber, M., Dicks, A. & Akçay, E. C4 photosynthesis and climate through the lens of optimality. *Proceedings of the National Academy of Sciences*, 115, 12057-12062 (2018).
2. Farquhar, G. D., Von Caemmerer, S. & Berry, J. A biochemical model of photosynthetic carbon dioxide assimilation in leaves of 3-carbon pathway species. *Planta* 149(1):78–90 (1980).

3. Von Caemmerer, S. Biochemical models of photosynthesis. In *Techniques in Plant Sciences* p. 196. CSIRO Publishing, Colingwood, Australia (2000).
4. Givnish, T. J. Optimal stomatal conductance, allocation of energy between leaves and roots, and the marginal cost of transpiration. In: *On the Economy of Plant Form and Function* (ed. Givnish TJ), pp. 171-213. Cambridge University Press, Cambridge (1986).
5. Tezara, W., Mitchell, V. J., Driscoll, S. D. & Lawlor, D.W. Water stress inhibits plant photosynthesis by decreasing coupling factor and ATP. *Nature* 401(6756):914-917 (1999).
6. Lawlor, D. W. & Cornic, G. Photosynthetic carbon assimilation and associated metabolism in relation to water deficits in higher plants. *Plant Cell & Environment* 25(2): 275-294 (2002).
7. Tang, A. C. Photosynthetic oxygen evolution at low water potential in leaf discs lacking an epidermis. *Annals of Botany* 89(7):861-870 (2002).
8. Vico, G. & Porporato, A. Modelling C_3 and C_4 photosynthesis under water-stressed conditions. *Plant and Soil* 313: 187-203 (2008).
9. Ögren, E. & Evans, J. R. Photosynthetic light response curves.1.The influence of CO_2 partial pressure and leaf inversion. *Planta* 189(2):182-190 (1993).
10. Wullschleger, S. D. Biochemical limitations to carbon assimilation in C3 plants—a retrospective analysis of the A/Ci curves from 109 species. *Journal of experimental botany* 44(5), 907-920 (1993).
11. Zhou, H., Akçay, E. & Helliker, B. Optimal coordination and reorganization of photosynthetic properties in C4 grasses. *Plant, Cell & Environment* 46(3), pp.796-811 (2023).
12. Leuning, R. A critical-appraisal of a combined stomatal photosynthesis model for C3 plants. *Plant Cell & Environment* 18(4):339-355 (1995).
13. Boyd, R. A., Gandin, A. & Cousins, A. B. Temperature responses of C_4 photosynthesis: biochemical analysis of Rubisco, phosphoenolpyruvate carboxylase, and carbonic anhydrase in Setaria viridis. *Plant Physiology* 169, 1850–1861 (2015).
14. Chen, D. X., Coughenour, M. B., Knapp, A. K. & Owensby, C. E. Mathematical simulation of C_4 grass photosynthesis in ambient and elevated CO_2. *Ecological Modeling* 73, 63–80 (1994).
15. Cousins, A. B., Ghannoum, O., von Caemmerer, S. & Badger, M. R. Simultaneous determination of Rubisco carboxylase and oxygenase kinetic parameters in *Triticum aestivum* and *Zea mays* using membrane inlet mass spectrometry. *Plant Cell & Environment* 33, 444–452 (2010).
16. Kubien, D. S., Whitney, S. M., Moore, P. V. & Jesson, L. K. The biochemistry of Rubisco in Flaveria. *Journal of Experimental Botany* 59, 1767–1777 (2008).
17. Pedomo, J. A., Cavanagh, A. P., Kubien, D. S. & Galmes, J. Temperature dependence of *in vitro* Rubisco kinetics in species of *Flaveria* with different photosynthetic mechanisms. *Photosynthesis Research* 124, 67–75 (2015).
18. Sharwood, R. E., Ghannoum, O., Kapralov, M. V., Gunn, L. H. & Whitney, S. M. Temperature responses of Rubisco from Paniceae grasses provide opportunities for improving C_3 photosynthesis. *Nature Plants* 2, 16186 (2016).
19. Sharwood, R. E., Ghannoum, O. & Whitney, S. M. Prospects for improving CO_2 fixation in C_3-crops through understanding C_4-Rubisco biogenesis and catalytic diversity. *Current Opinion in Plant Biology* 31, 135-142 (2016).

20. Ubierna, N., Gandin, A., Boyd, R. A. & Cousins, A. B. Temperature response of mesophyll conductance in three C_4 species calculated with two methods: ^{18}O discrimination and in vitro V_{pmax}. *New Phytologist* 214, 66-80 (2017).
21. Sonawane, B. V., Koteyeva, N. K., Johnson, D. M. & Cousins, A. B. Differences in leaf anatomy determines temperature response of leaf hydraulic and mesophyll CO2 conductance in phylogenetically related C4 and C3 grass species. *New Phytologist* 230(5), 1802-1814 (2021).
22. Pengelly J. J., Sirault X. R., Tazoe Y., Evans J. R., Furbank R. T. & von Caemmerer S. (2010). Growth of the C_4 dicot *Flaveria bidentis*: photosynthetic acclimation to low light through shifts in leaf anatomy and biochemistry. *Journal of Experimental Botany* 61, 4109-4122.
23. Yin, X., Van Der Putten, P. E., Driever, S. M. & Struik, PC. Temperature response of bundle-sheath conductance in maize leaves. *Journal of Experimental Botany* 67, 2699-2714 (2016).
24. Bernacchi, C. J., Singsaas, E. L., Pimentel, C., Portis, A. R. & Long, S. P. Improved temperature response functions for models of Rubisco-limited photosynthesis. *Plant Cell & Environment* 24(2), 253-259 (2001).
25. Bernacchi, C. J., Pimentel, C. & Long, S. P. In vivo temperature response functions of parameters required to model RuBP-limited photosynthesis. *Plant Cell & Environment* 26(9), 1419- 1430 (2003).
26. Massad, R. S., Tuzet, A. & Bethenod, O. The effect of temperature on C_4-type leaf photosynthesis parameters. *Plant Cell & Environment* 30, 109-122 (2007).
27. Bernacchi, C. J., Portis, A. R., Nakano, H., von Caemmerer, S. & Long, S. P. Temperature response of mesophyll conductance. Implications for the determination of Rubisco enzyme kinetics and for limitations to photosynthesis in vivo. *Plant Physiology* 130(4), 1992-1998 (2002).
28. Pinto, H., Sharwood, R. E., Tissue, D. T. & Ghannoum, O. Photosynthesis of C_3, C_3–C_4, and C_4 grasses at glacial CO_2. *Journal of Experimental Botany* 65, 3669-3681 (2014).
29. Taylor, S. H., Hulme, S. P., Rees, M, Ripley, B. S., Ian Woodward, F. & Osborne, C. P. Ecophysiological traits in C_3 and C_4 grasses: a phylogenetically controlled screening experiment. *New Phytologist 185*(3), 780-791 (2010).
30. Taylor, S. H., Ripley, B. S., Martin, T., De-Wet, L. A., Woodward, F. I. & Osborne, C. P. Physiological advantages of C_4 grasses in the field: a comparative experiment demonstrating the importance of drought. *Global Change Biology 20*(6), 1992-2003 (2014).
31. Zhou, H., Akçay, E., Edwards, E. & Helliker, B. R. The legacy of C_4 evolution in the hydraulics of C_3 and C4 grasses. *bioRxiv*. doi: https://doi.org/10.1101/2020.05.14.097030 (2020).
32. Ubierna, N., Sun, W., Kramer, D. M. & Cousins, A. B. The efficiency of C_4 photosynthesis under low light conditions in *Zea mays*, *Miscanthus* X *giganteus* and *Flaveria bidentis*. *Plant Cell & Environment* 36, 365-381 (2013).
