# Supplementary material for: Mapping the global distribution of C4 vegetation using observations and optimality theory
Source: Nat Commun. 2024 Feb 9;15:1219. doi: 10.1038/s41467-024-45606-3 (PMC10858286; doi:10.1038/s41467-024-45606-3)
Supplement: Supplementary file 3 — Reporting Summary [file 41467_2024_45606_MOESM3_ESM.pdf]

nature portfolio

Corresponding author(s): Xiangzhong Luo

Last updated by author(s): Jan 16, 2024

Reporting Summary

Nature Portfolio wishes to improve the reproducibility of the work that we publish. This form provides structure for consistency and transparency in reporting. For further information on Nature Portfolio policies, see our Editorial Policies and the Editorial Policy Checklist.

Statistics

For all statistical analyses, confirm that the following items are present in the figure legend, table legend, main text, or Methods section.

n/a	Confirmed	
☐	☒	The exact sample size (*n*) for each experimental group/condition, given as a discrete number and unit of measurement
☒	☐	A statement on whether measurements were taken from distinct samples or whether the same sample was measured repeatedly
☒	☐	The statistical test(s) used AND whether they are one- or two-sided<br>*Only common tests should be described solely by name; describe more complex techniques in the Methods section.*
☐	☒	A description of all covariates tested
☐	☒	A description of any assumptions or corrections, such as tests of normality and adjustment for multiple comparisons
☐	☒	A full description of the statistical parameters including central tendency (e.g. means) or other basic estimates (e.g. regression coefficient) AND variation (e.g. standard deviation) or associated estimates of uncertainty (e.g. confidence intervals)
☐	☒	For null hypothesis testing, the test statistic (e.g. *F*, *t*, *r*) with confidence intervals, effect sizes, degrees of freedom and *P* value noted<br>*Give P values as exact values whenever suitable.*
☒	☐	For Bayesian analysis, information on the choice of priors and Markov chain Monte Carlo settings
☐	☒	For hierarchical and complex designs, identification of the appropriate level for tests and full reporting of outcomes
☒	☐	Estimates of effect sizes (e.g. Cohen's *d*, Pearson's *r*), indicating how they were calculated

Our web collection on statistics for biologists contains articles on many of the points above.

Software and code

Policy information about availability of computer code

Data collection	We did not use software and code for data collection.
Data analysis	The code for analysis is available at https://github.com/lxzswr/C4distribution/ and https://zenodo.org/records/10516423. The code of optimality photosynthesis model is available at https://github.com/zhouhaoran06/C3C4OptPhotosynthesis-.

For manuscripts utilizing custom algorithms or software that are central to the research but not yet described in published literature, software must be made available to editors and reviewers. We strongly encourage code deposition in a community repository (e.g. GitHub). See the Nature Portfolio guidelines for submitting code & software for further information.

Data

Policy information about availability of data

All manuscripts must include a data availability statement. This statement should provide the following information, where applicable:

- Accession codes, unique identifiers, or web links for publicly available datasets
- A description of any restrictions on data availability
- For clinical datasets or third party data, please ensure that the statement adheres to our policy

The global C4 vegetation distribution map is available at https://zenodo.org/records/10516423. The CS C4 map was acquired from https://daac.ornl.gov/cgi-bin/dsviewer.pl?ds_id=932. The CRU TS4.02 climate data is available at https://crudata.uea.ac.uk/cru/data/hrg/, the soil moisture data can be downloaded from https://esa-soilmoisture-cci.org/, browsing for version 07. The global dataset of leaf photosynthetic pathway was acquired from the TRY database https://www.try-db.org/

TryWeb/Home.php, by selecting those records with the field "photosynthesis pathway (traitID: 22)". The DG dataset was obtained from the supporting information of https://onlinelibrary.wiley.com/doi/10.1111/jbi.13061. The subset of the observations from the nutrient network (NutNet) are accessible at https://portal.edirepository.org/nis/mapbrowse?packageid=edi.1037.2.

Human research participants

Policy information about studies involving human research participants and Sex and Gender in Research.

Reporting on sex and gender	NA
Population characteristics	NA
Recruitment	NA
Ethics oversight	NA

Note that full information on the approval of the study protocol must also be provided in the manuscript.

Field-specific reporting

Please select the one below that is the best fit for your research. If you are not sure, read the appropriate sections before making your selection.

☐ Life sciences ☐ Behavioural & social sciences ☒ Ecological, evolutionary & environmental sciences

For a reference copy of the document with all sections, see nature.com/documents/nr-reporting-summary-flat.pdf

Ecological, evolutionary & environmental sciences study design

All studies must disclose on these points even when the disclosure is negative.

Study description

We used few open leaf trait databases, in combination with remote sensing observations and a photosynthetic optimality model to provide a novel estimate of global C4 distribution, and investigate the reasons for C4 change in the past few decades.

Research sample

We used all the available records of C4 from the open trait TRY database for the analysis. We used multiple land use maps based on different remote sensing data to get a good quality global herbaceous fraction from 2000 to 2019.

Sampling strategy

We acquired 61588 records of photosynthetic pathways from the TRY database (last accessed 2022 June), among them, there were 2269 records of C4. We first removed the woody species from the records, based on species names and an index table from the TRY database. The rationale of the step is that the majority of C4 species are the non-woody type. After the step, we kept 13919 records for non-woody species, among which 1881 were C4 natural grass species. We then aggregate these records to 10x10 degree cells, in each cell we calculate the species abundance of C4 (i.e., number of C4 species/total number of herbaceous species). The gridded values of C4 species abundance would be further used to constrain the optimality model to estimate global C4 abundance. Here we use the large-size grid cell to make sure there were enough samples in cells to acquire a meaningful estimate of C4 abundance – in this analysis, each cell has at least records.

To acquire C4 area abundance from C4 species abundance, we used an open dataset from the global nutrient network that has paired C4 species abundance and C4 area abundance to infer their relationship . The dataset includes species-specific coverage records as well as the grass species richness data collected in 25 m2 plots across 34 sites. Each site has between 1 and 6 control plots. Note that we only used the data from the control plots, excluding plots that underwent nutrient addition treatments.

Data collection

Data were directly downloaded by the lead author using the links provided in the data availability statement during the research period 2022 - 2023.

Timing and spatial scale

The study is based on a wide array of secondary datasets, we only need to access the datasets once. There is no need to deploy sampling strategies. As for the output, we provide annual estimate of global C4 distribution, including C4 natural grasses and C4 crops , at a 0.5 degree grid.

Data exclusions

We did not perform data exclusion in the analysis.

Reproducibility

We have released the code to support the reproduction of the figures in the manuscript. The new global C4 distribution map is available at https://zenodo.org/records/10516423, which can also be used to produce our results.

Randomization

We did not group data in most parts of the analysis. Only when investigating the relationship between C4 area abundance and C4 species richness, to avoid the uneven distribution of data samples, we grouped the paired observations by their C4 species richness, and for each species abundance we get a mean C4 area abundance and the standard deviation of the C4 area abundance. We then conducted 1000 linear fittings (i.e., with an intercept of 0, since C4 area abundance should be 0 when C4 species abundance is 0), and for each fitting we used randomly sampled C4 area abundance values (i.e., based on mean and the standard deviation) value against C4 species richness values. The slopes of the linear regressions represented a conversion factor between C4 species richness

	and C4 area abundance.
Blinding	Blinding is not relevant in our study. All the raw data used in the study are previously published and open-access.

Did the study involve field work? ☐ Yes ☒ No

Reporting for specific materials, systems and methods

We require information from authors about some types of materials, experimental systems and methods used in many studies. Here, indicate whether each material, system or method listed is relevant to your study. If you are not sure if a list item applies to your research, read the appropriate section before selecting a response.

Materials & experimental systems

n/a	Involved in the study
☒	☐ Antibodies
☒	☐ Eukaryotic cell lines
☒	☐ Palaeontology and archaeology
☒	☐ Animals and other organisms
☒	☐ Clinical data
☒	☐ Dual use research of concern

Methods

n/a	Involved in the study
☒	☐ ChIP-seq
☒	☐ Flow cytometry
☒	☐ MRI-based neuroimaging
